# Nigeria's energy review: Focusing on solar energy potential and penetration

**DOI:** 10.1007/s10668-022-02308-4

**Published:** 2022-04-13

**Authors:** Yusuf. N. Chanchangi, Flossie Adu, Aritra Ghosh, Senthilarasu Sundaram, Tapas. K. Mallick

**Affiliations:** Environment and Sustainability Institute (ESI), University of Exeter, Penryn Campus, Penryn, TR10 9FE UK

**Keywords:** Nigeria, Energy, Electricity, Renewable energy, Solar energy, Potential and penetration, Electricity crisis

## Abstract

In Nigeria, the rapid population increase and the overreliance on fossil fuel have created significant environmental, health, political, and economic consequences leading to severe socio-economic drawbacks. These factors have developed a wide gap between energy demand and supply due to insufficient local production, necessitating a clean energy supply for all. The photovoltaic device's economic and environmental merits have made it the most suitable clean energy alternative to help developing countries such as Nigeria achieve the SDG-7. However, apart from the device's low efficiency, which is undergoing intensive study globally, other factors affect the penetration of the technology in developing countries, particularly Nigeria. This report systematically reviews the literature on the country's energy crisis and renewable energy potential, leading to an overview of solar energy potential and penetration. The potential of the technology and its penetration in the country were provided. A list highlighting challenges hindering technology penetration was also provided, and a solution for each was recommended.

## Introduction

The rapid increase of the world population and present-day industrial development and lifestyle have created a massive demand and energy supply gap. This gap demands a rapid increase of substantial clean, stable, and reliable energy supply. The over-dependency on depleting fossil fuel in generating energy for our daily demand increasingly affects our environment and deteriorates human health. Access to stable and reliable energy is still a dream for most developing countries, and its deficiency is ominous for development, as energy access is crucial to economic development, socio-economic activities, agricultural activities, and living standard. According to Akinyele et al. (2015); Sanusi and Owoyele (2016); Baurzhan and Jenkins (2016); and Monyei et al. (2017), about 1.2 billion individuals are still living without access to modern (stable) energy, with about 50% of them residing in sub-Saharan Africa. Nigeria, the country with the highest population in the region, has about 100 million citizens living without clean and stable energy (Akinyele et al., 2017; Yakubu & Ifeanyi-Nwaoha, 2017). This signifies that the country is suffering from acute energy poverty.

Nigeria is endowed with conventional energy resources (Non-Renewable) and renewable energy resources (Biomass, Hydro, Solar, and Wind) that are sufficient to satisfy the demand of its populace and export the excess to neighbouring countries as a tradable commodity to generate funds. Thus far, the in-house supply is still inadequate and unable to meet the country's demand, and this is continuously increasing due to a continuous increase in population, requiring an immediate response before it reaches an unrecoverable situation. This inadequacy of supply has several consequences. The Nigerian government has disbursed billions of dollars over the last two decades to improve and boost energy supply using conventional energy resources, yet the country is still plagued with severe energy shortages (Usman et al., 2015). The numerical value relating to some investments is provided in this report's subsequent section. This energy supply shortage poses challenges to both the rural and urban populations. However, the problem is most severe in the rural areas, where the majority of the population lives without access to the national grid, and besides, even the majority of urban dwellers suffer from an unstable and insufficient power supply. The frequent power outages have compelled many Nigerians to adopt self-energy generation using various fossil fuel-powered generators to generate electricity for domestic, commercial, and industrial consumption. The by-products of this have adverse effects on humans and the environment.

Energy deficit leads to poverty, economic decline, low standard of living, citizen hardship, and many more negative impacts. Conversely, a constant and stable energy supply is fundamental to the development of a nation. According to Ikem et al. (2016), energy supply is the pivot supporting the development wheel. Ayodele and Ogunjuyigbe (2015); Oseni (2012); Oyedepo (2012a); Rafindad (2016), Anumaka (2012), and Akuru et al. (2017) established that stable and reliable energy (electricity) supply is exceptionally significant and it is an essential ingredient and a necessary tool that plays a vital role in the economic development, poverty reduction, industrial, agriculture, manufacturing, commerce, infrastructural development, employment, and security. It also plays a vital role in ensuring that basic needs and services (food and water, housing, health services, and education) are adequately provided. Anumaka (2012) stated that the contemporary world depends on abundant energy for developmental growth.

Moreover, Nigeria's energy poverty is very alarming (Edomah et al., 2021), with some reports describing the electricity's performance records as appalling (Ebhota & Tabakov, 2018); thus, it becomes imperative for unceasing research on the energy situation to provide possible solutions to mitigate the problem. Worldbank (2021) also stated that many people live without access to the grid, making the situation very concerning, affecting the citizens and economic growth of the country. Several types of research have been conducted to explore the potential of clean and reliable energy in Nigeria. Sambo (2009) stated that clean energy is the solution for Nigeria's acute energy crisis, predominantly in rural areas. Ajayi and Ajayi (2013) stated that fossil fuel by-products are harmful to humans and the deleterious environment. Aliyu et al. (2015) also believe that there is a need to rigorously pursue renewable energy technology to conquer Nigeria's continuing energy deficit to progress. Such renewable energies as clean energy should be considered viable options for sustainable electricity production in Nigeria.

As earlier stated, rapid population growth has made the energy demand increase beyond the supply in Nigeria. The country has suffered from this energy deficit for over two decades. The majority of the populace does not have access to the national utility grid and are left with the option of self-energy generation using firewood and/or fossil fuel that significantly affects both the environment and its inhabitants. The exploitation of renewable energy to compensate for the rapid increase of population, especially in the country's remote areas, could be a solution (Mohammed et al., 2013). Several prior studies have established that a sufficient and stable energy supply is vital to any country's development. Without the minimum required access to energy for many of its population, a nation cannot develop and sustain itself beyond a subsistence economy.

Prior studies show that renewable energy can resolve the persisting energy crisis in the country that has lasted for over two decades. Some of these research papers are as follows: Shaaban and Petinrin (2014) concluded that the country is suffering from an acute electricity deficit and advocate that the use of renewable energy can solve the persisting energy crisis in the country by bridging the demand and supply gap as well improving the living standards of the people residing in the rural areas. Ohunakin (2010) studied the current and future perspectives on renewable energy application in Nigeria. The research concluded that energy demand is very high and continuously increasing because of the continuous increase in the population. The researcher further recommended exploiting the renewable energies available and diversifying the energy supply. Anumaka (2012) concluded that the Nigerian government alone could not provide adequate funding to supply electricity. They recommended that private and foreign investors are required to promote the integration of renewable energy to minimise low access to electricity in rural areas. Mohammed et al. (2013) concluded that renewable energy could serve as a solution to the energy poverty in the rural areas and further recommended that in order to keep with the pace of rapid population growth in the country, there is a need to exploit renewable energy to compensate for the energy deficit.

Ajayi et al. (2016) conducted an assessment of solar and wind resources' potentials in Northern Nigerian and concluded that solar photovoltaic technology is a viable option to facilitate sustainable development goals. Akimbami (2001) conducted an analytical review of renewable energy policies in Nigerian. The researcher concluded that renewable energy resources could decentralise energy supply and increase energy security. Akuru et al. (2017) highlighted the potential of renewable energy in Nigeria and concluded that a 100% renewable energy supply is possible in Nigeria because it has already been achieved elsewhere; however, it requires a substantial financial commitment. As reported by Dodondawa (2019) and Daily_Trust (2019), the Managing Director of Shell Nigeria Exploration and Production Company (SNEPCO), Mr Bayo Ojulari, stated that Nigeria requires approximately USD 40 billion to USD 200 billion so as to close the energy gap which would provide 30 gigawatts (GW) to 175 GW generation capacity. However, this estimation does specify if the generation is renewable or not but is exceptionally huge.

Aliyu et al. (2015) reviewed and presented the current status of Nigeria's primary renewable energies and their potentials. They recommended and promoted the integration of renewable energy in the supply mix. Ikem et al. (2016) presented an overview of the Nigerian national electricity grid, concluded an inadequate flow of information regarding renewable energy, and recommended that the Nigerian government pursue the renewable energy policy yet implemented. The research further stated that renewable energy resources could also increase reliable and stable supply, sustaining its growth. Sambo (2009) presented the role of renewables in bridging the energy demand and supply gap. This prominent researcher in Nigeria concluded that renewable energy is a viable option to solve the country's persisting energy crisis.

This study aims to provide a comprehensive insight into Nigeria's electricity scenario and highlight the potential of renewable and non-renewable sources in the region with the challenges hindering their penetration, furthermore, to recommend a solution that could be used as a foundation for conducting further scientific and social/political science studies for the country's development and substance of renewable energy section.

There are about 14 types of review, and each comes with methodologies that prescribe a critical scientific and philosophical approach known as Search, Appraisal, Synthesis, and Analysis (SALSA) to yield a high standard quality report that could contribute to the advancement of research and development (Grant and Booth, 2009). This study adopted systematic search and review, combining comprehensive search with a critical assessment with minimal narrative to provide recommendations based on the synthesised evidence. This particular review system attempts to acquire broad knowledge in the area of study provide insight about it rather than resolving or answering a complex search. The approach used search engines such as Google, Google Scholar, IEEE Xplore, Scopus, ScienceDirect, and ResearchGate database. Keywords such as Nigeria, energy in Nigeria, electricity in Nigeria, renewable energy in Nigeria, solar energy in Nigeria, RE potential and penetration in Nigeria, electricity crisis in Nigeria, and factors influencing electricity crisis in Nigeria, challenges causing lower RE penetration in Nigeria were used to search on the various databases mentioned above. A broader time scope of over two decades from 1999 to 2021 was considered during the search with a comparative analysis with the most recent papers to identify disparities.

## Brief historical background of electricity in Nigeria

Historically, electricity generation in Nigeria can be traced to1896 in Lagos state, 15 (fifteen) years after it was first produced in England (Anumaka, 2012; Oseni, 2012). In 1929, Nigeria Electricity Supply Company (NESCO) commenced its construction operations of a hydropower plant in Jos, Plateau state, in the Northern part of the country (Kumar & Nagarajan, 2016; Monyei et al., 2017). In 1951, the colonial legislative council established the Electricity Corporation of Nigeria (ECN) through parliament. The body was established to manage electricity supply and development (Anumaka, 2012; Monyei et al., 2017; Vincent & Yusuf, 2014). In 1962, expansion led to the Niger Dam Authority (NDA) development through the Nigerian parliament's act to construct and maintain dams for electricity generation, water supply, agriculture, and irrigation activities (Anumaka, 2012). The agreement at that time was all energy produced by NDA would be sold to ECN.

In 1972, Nigerian Electricity Power Authority (NEPA) was established, ECN and NDA were merged to form NEPA, and the Authority was mandated to control and manage the entire activities of the power sector (Anumaka, 2012). NEPA monopolised the sector for over 3 (three) decades (1972–2005). It managed both the country's generation, transmission, and electricity distribution. With the rapid increase of population, the demand continued to increase, and it reached a stage where the demand superseded the supply coupled with dilapidated infrastructure and unqualified workforce; NEPA was finding difficulties in managing the sector. Therefore, in 1979, the Energy Commission of Nigeria (ECN) was established through parliament with the statutory mandate to strategically plan and coordinate national energy policy (Anumaka, 2012).

In 2001, National Electric Power Policy ( NEPP) was introduced, which led to the power sector revolution. This policy developed an Electric Power Sector Reform (EPSR) documented in the same year, but the document was not signed until 2005 (Vincent & Yusuf, 2014). After this process, NEPA was transformed and renamed Power Holding Company of Nigeria (PHCN). In the same year, the president signed the Power Sector Reform Bill law and established the Nigerian Electricity Regulatory Commission (NERC) to regulate energy sector activities, issue licences to stakeholders, determine and regulate tariffs, and enforce standards. The commission enabled the unbundling of the sector and involvement of the private sectors in both generation and distribution, but this privatisation process was delayed by union agitation and protests until 2006 (Anumaka, 2012). In 2005, NERC was also established Niger Delta Power Holding Company Limited (NDPHC) as a limited liability entity to manage the National Integrated power projects (NIPP) in the Niger Delta region (Usman et al., 2015).

In 2010, Nigeria Bulk Electricity Trading (NBET) PLC was established through an act of parliament with a mandate to serve as a broker between distribution companies and Independent Power Producers (IPP) (Usman et al., 2015). The federal government also established the Nigerian Electricity Liability Management Company (NELMCO) to oversee the assets and liabilities of the PHCN in the same year; another reform called the Power Sector Roadmap was lofted (Monyei et al., 2017). Finally, privatisation of energy generation and distribution was completed in 2014, and companies were handed over to respective purchasers accordingly (Usman et al., 2015). In 2015, the federal government approved National Renewable Energy and Energy Efficiency Policy (NREEEP). This policy intends to integrate renewable energy into the national supply mix to increase electricity access to the population (Yakubu et al. 2017).

### Overview of key statistical indicators

#### 2.1.1 Country context

Nigeria is a sub-Saharan country located in West Africa on the Gulf of Guinea. The country has a total surface area of 923 768. 00 km^2^ comprised of about 1.4% water area (13,000 km^2^) and about 98.6% land area (910,768 km^2^) (Ajayi et al., 2016; Akinyele et al., 2017; UN, 2017). The country lies between latitudes 4.321 N and 14.1 N of the equator and longitude 2.721E and 14.641E to the Greenwich meridian (Ajayi et al., 2016; Akinyele et al., 2015). It has a varied terrain including several hills, mountains, rivers, lakes, forests, and plains scattered and varies from semi-desert and savannah in the North to tropical forest and coastal swamp in the Southern part (Anumaka, 2012; Shaaban & Petinrin, 2014). The country has 78% of agricultural land that comprises 37.3% of arable land, 7.4% of permanent crops, and 33.3% of permanent pastures. It also has a forest land area of 9.5% and others 12.5%. The country's irrigated land area is about 2930 km^2^ (CIA, 2018). Nigeria is the 7^th^ most populous nation globally, with an estimated 200.96 million in 2019 (World-Bank, 2021). The nation's income is highly dependent on crude oil, with about 90% of Nigeria's income related to crude oil activity.

Meteorologically, the country enjoys two tropical climatic conditions, the wet and dry seasons. These seasonal durations differ by region: the Southern part experiences a more extended rainy season from March to December with a maximum of 2600 mm/yr, and December to March is the dry season. The Southern part is usually wet and hot. On the other hand, the Northern part is usually dry and hot, with an average temperature range between 32 °C and 42 °C. The rainy season in Northern Nigeria commences around April and lasts till October with about 110 mm/yr, and the dry season is from October to April and is accompanied by dry, dusty wind (Harmattan) (Giwa et al., 2017; Oji et al., 2012; Shaaban & Petinrin, 2014).

#### Nigeria's electricity infrastructure

**Fig. 1:**
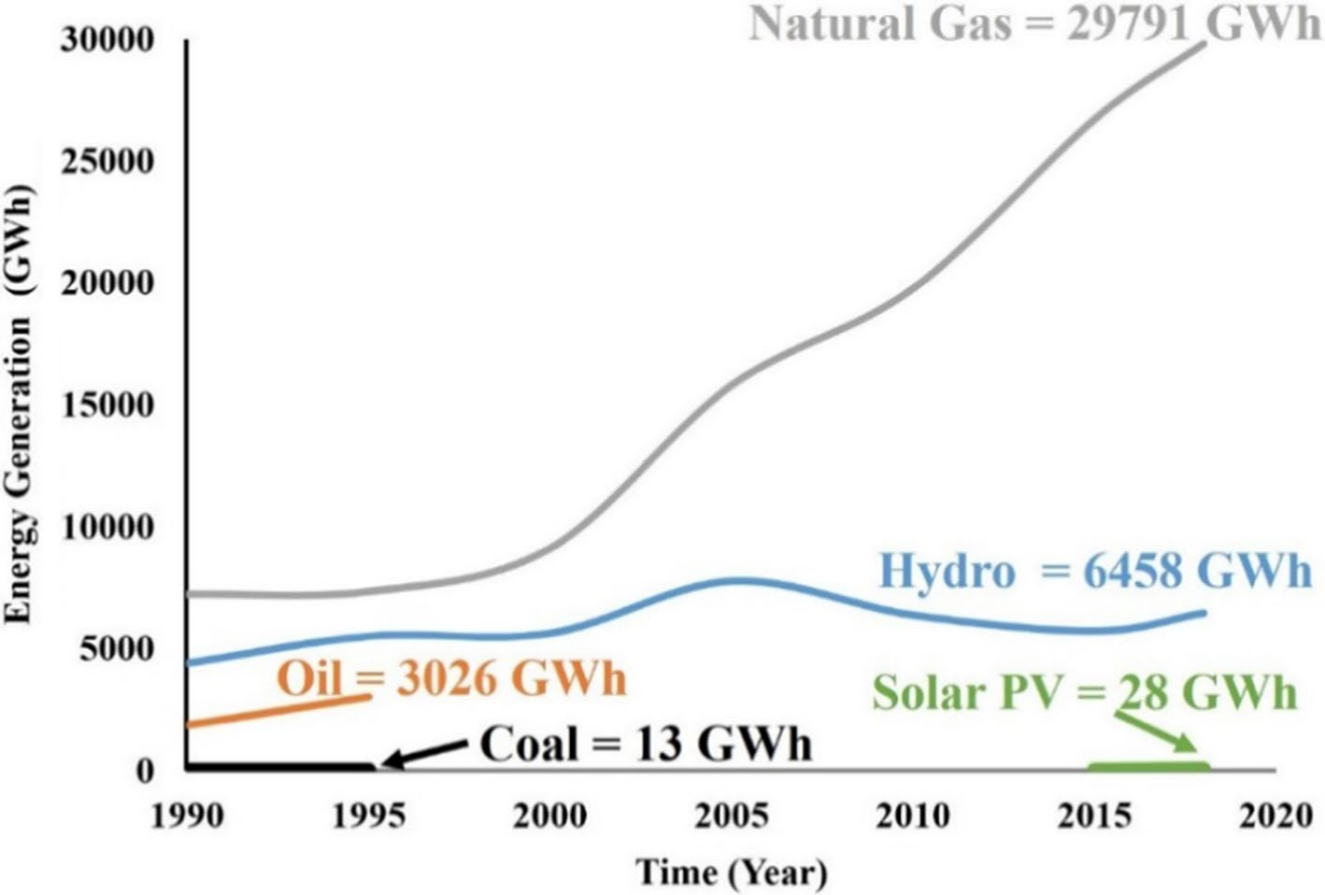
Variation of energy generation and its sources for over three decades (IEA, 2021a, 2021b, 2021c)

Nigeria is a country that has access to abundant non-renewable and renewable resources capable of harvesting electricity. However, electricity generation to meet up with the demand has been one of the country's significant challenges. According to Akinyele et al. (2017), Nigeria's electricity infrastructure's low status has led to increased use of fossil fuel to power generators by a large number of Nigerians to meet their daily needs. This problem has left many Nigerians without an option other than to adopt self-energy generation using fossil fuel-powered generators and traditional biomass. According to Anumaka (2012), Nigeria's electricity grid generation has been dominated by gas and other oil, hydroelectric, and coal sources. In 2014, Nigeria produced around 30 TWh of electricity distributed in the following manner: Oil 6 TWh, Hydroelectricity 6 TWh, Gas 18 TWh, solar energy 0.028 TWh (IEA, 2021a, 2021b, 2021c). This distribution is shown in Fig. 1.

#### Sub-sectors

**Fig. 2:**
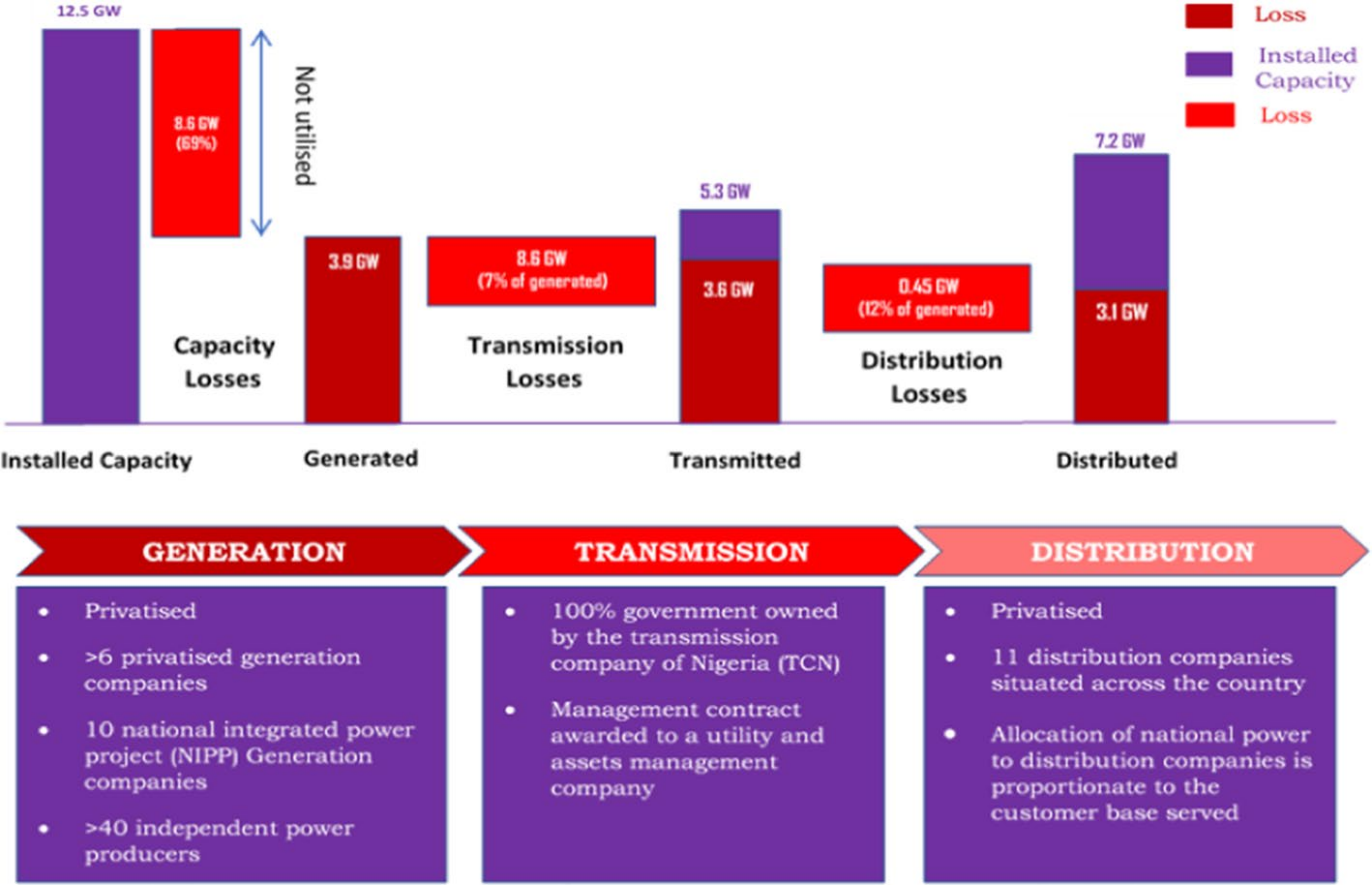
Summary of Nigeria's electricity system ( adopted from Wijeratne et al. (2016))

Nigeria's electricity system is divided into three sub-sectors: generation, transmission, and distribution. These sectors currently have 23 (twenty-three) grid-connected energy generation plants (hydro and thermal) controlled by the government and private sectors. The sector has a transmission company with an extensive network connection around the country and is managed by the government alone. The distribution sector has 11 (eleven) companies that manage distribution to consumers that are owned and managed by private companies. These sub-sectors are described in detail in the subsequent section and summarised in Fig. 2.

Several researchers have reported that as of December 2013, Nigeria had a total installed capacity of about 10,396 MW from 23 power generating stations across the country, out of which 8457.6 MW is from the thermal-based station with an available capacity of 4996 MW (Bassey-Etuk & Ifeanyi-Nwaoha, 2017; Ikem et al., 2016; Vincent & Yusuf, 2014). Also, the hydro-based power station accounts for about 1938 MW to 1060 MW as its available capacity. These 23 power plants are owned and managed by both government (National Integrated Power Projects (NIPP) and private companies (Independent Power Producers and privatised Generation Companies (IPP)), with the government still having the majority of the share (Bassey-Etuk & Ifeanyi-Nwaoha, 2017; Ikem et al., 2016; Vincent & Yusuf, 2014).

Figure 2 shows a summary of the power sector in 2015. Wijeratne et al. (2016) reported that in 2015, the country had an installed capacity of about 12,500 MW with about 3900 MW available capacities, and 3100 MW is supplied. In 2017, the total installed capacity increased to about 14,380 MW from 27 generating plants across the country, out of which only about 7527.5 MW was available and between 3000 to 5000 MW is supplied (Akuru et al., 2017). According to National Bureau for Statistics (NBS) (NBS, 2017), the peak generation was attained on 24 January 2017, where about 5846 MW (140,316 MWh) was generated, and about 5747 MW (137,920 MWh) was supplied. This shows remarkable improvement in the sector. However, the supply is still inadequate, and more alternative clean sources must be harnessed.

The power transmission is solely managed by the Transmission Company of Nigeria, owned by the government, characterised by power losses and dilapidated infrastructures. The transmission system has a network of about 12,300 km in length with about 5523.8 km of 330 kV lines and 6801.49 km of 132 kV lines (Ikem et al., 2016; Usman et al., 2015; Vincent & Yusuf, 2014). There are 137 transmission stations across the country consisting of 32 330/132 kV substations with an installed capacity to transform 7688 MVA. The remaining 105 are 132/33/11 kV substations with an installed capacity of 9130 MVA (Vincent & Yusuf, 2014).

Although the country has a huge energy deficit, it is constrained to an agreement to export part of its generated electricity to neighbouring West African countries such as Niger and Benin republics (Adeoye and Spataru, 2020). About 395 km of 132 kV transmission line goes from Nigeria to Niger republic and additional 255 km of 330 kV transmission line proposed by Economic Community of West African States (ECOWAS) before 2030 (Adeoye and Spataru, 2020). Similarly, about 70 km of 330 kV transmission line stretched from Nigeria to the Benin republic (Adeoye and Spataru, 2020).

In recent years, the government has been improving the transmission network and its infrastructure. Transmission and distribution (T&D) losses are drastically dropping but are still considered high. In 2013, Nigeria recorded losses of about 35% (Ikem et al., 2016), while in 2014, the country recorded about 16.107% losses through transmission and distribution (WorldBank, 2017a). These losses dropped to about 8% in 2016, the Multi-Year Tariff Order (MYTO) target. Additional projects to expand the transmission network are ongoing, and it is expected to extend the network with about 986 km of 330 kV and 705 km of 132 kV.

Distribution is the third sub-sector of the power system, which interacts with the consumers. The sector comprises 11 electricity distribution companies (DISCOs) with their various load allocations. Their distribution capacity is only around 5000 MW to 6000 MW because they inherited old assets, and about 40% of them have been more than 50 years old in operation. Poor electricity distribution is a significant contributor to T&D (Transmission and Distribution) losses (Ezeokoli & Ifeanyi-Nwaoha, 2018). The substation transformers at the distribution level receive the voltage at about 115 kV and convert it from 4 to 34 kV. In contrast, the line transformer converts the energy to primary voltage, typically about 240 V and 480 V. The distribution sector is facing challenges such as poor infrastructure, weak and inadequate coverage, inadequate working tools, obsolete communication technology, staff that lacks training, insufficient funding, and lack of maintenance (Vincent & Yusuf, 2014).

## Nigeria's energy scenario

Nigeria is facing severe challenges in electricity generation and supply. The national grid supply is mainly characterised by continuous fluctuations, frequent power outages, and system instability (Onohaebi & Eseosa, 2014). According to Oseni (2016), a typical Nigerian house has access to an average of five hours of electricity supply daily from the national grid. Ajayi and Ajayi (2013) stated that the national grid's Nigerian electricity production has not been sufficient to meet the country's demand, promoting self-generation using traditional biomass and fossil fuel. The country's electricity supply has sunk into a quagmire for over two decades. The supply is grossly insufficient and inadequate to meet the population's demand, continuously increasing; thus, the demand and supply gap is also rapidly widening. As stated earlier, Nigeria has an approximate population of over 200 million with an average estimated demand of 31.2 GW, and the country has an installed capacity of 14.38 GW with an average supply capacity of 6 GW (Akuru et al., 2017). It implies that the electricity generation system can only produce about 40% of its installed capacity. If the thumb rule, which says every 1,000,000 people require a minimum of 1000 MW, is applied to Nigeria, it will require about 200 000 MW (200 GW).

**Fig. 3:**
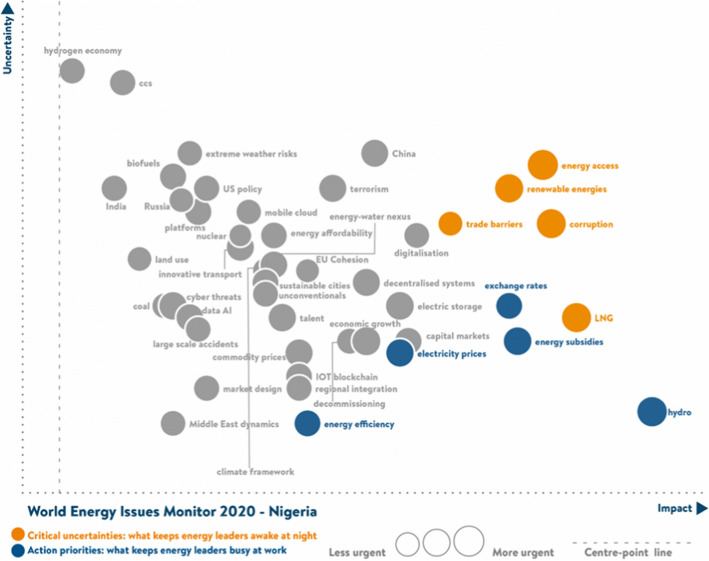
Energy uncertainty indicator (WEC, 2020)

Electricity production from renewable sources, excluding hydroelectric, is still 0% (WorldBank, 2017b). However, the status has recently changed with the aggressive development of solar energy installation. According to World Energy Council (WEC) (WEC, 2020), indicators, energy access, renewable energies, corruption, trade barriers, and liquefied natural gas, the critical uncertainty that Nigeria's leaders are struggling with; energy poverty, electricity prices, energy subsidy, and energy efficiency are significant uncertainties requiring government attention in 2020. Figure 3 shows the uncertain factors, priority level, impacts, and decoding signalling factors that can be obtained (WEC, 2020).

The government is making various attempts to resolve the energy crisis, but the progress is plodding. A considerable amount of money was invested in the sector in the last two decades, but a slight improvement has been recorded. Almost USD 20 billion was used to revive the Nigerian electricity sector, but the minimal effect (Newman, 2019). No one can appropriately account for the money. The investment was a policy intended to provide additional sources (renewable energy) such as solar photovoltaic installation in generation mix (WeForum, 2018); that could boost generation and reduce reliance on grid national grid through mini-grids and isolated areas. The power supply that is inadequate and unreliable continues to compel people to self-energy generation using traditional firewood's fossil fuel and use. These have serious negative repercussions on our health and the environment. A significant number of lives have been lost due to the emission of these fossil fuel generators, and quantitative figures in respect of the lives lost were presented in some reported accidents cited in the subsequent Sect. 3.1.1 of this report.

The insufficient supply of electricity and drop of the oil and gas prices in the international oil market has compelled and dragged Nigeria's economy into a recession. However, it has been projected that the country's economy will recover before 2021 (AfDB, 2017), but the economy is now reversing from recovery to bad status and worse than ever before, with the country declaring recession in 2020 and inflation rate reaching 11.4% in the first quarter of 2021 (AfDB, 2021).

The pandemic (SARS-CoV-2) also contributed to the country's economic decline. Adesina (2021) stated that the country recorded the lowest economic decline over two decades during the pandemic, about − 1.8% in 2020 and a − 1.8% decline of GDP in the same year. Anyanwu and Salami (2021) reported that a foreign direct investment (FDI) inflow declined from USD 3.3 billion in 2019 to USD 2.6 billion. African Markets Online (2020) indicates a − 3.37% decrease in the year‐to‐date stock exchange by 26 March 2021. This significantly affects the food security, health, and electricity sectors. Babatope and Soloman (2020) reported that the Covid 19 pandemic increased household demand and decreased industrial demand, leading to an aggregated drop in electricity demand. However, since the demand and supply gap is vast, the generation was inadequate.

**Fig. 4:**
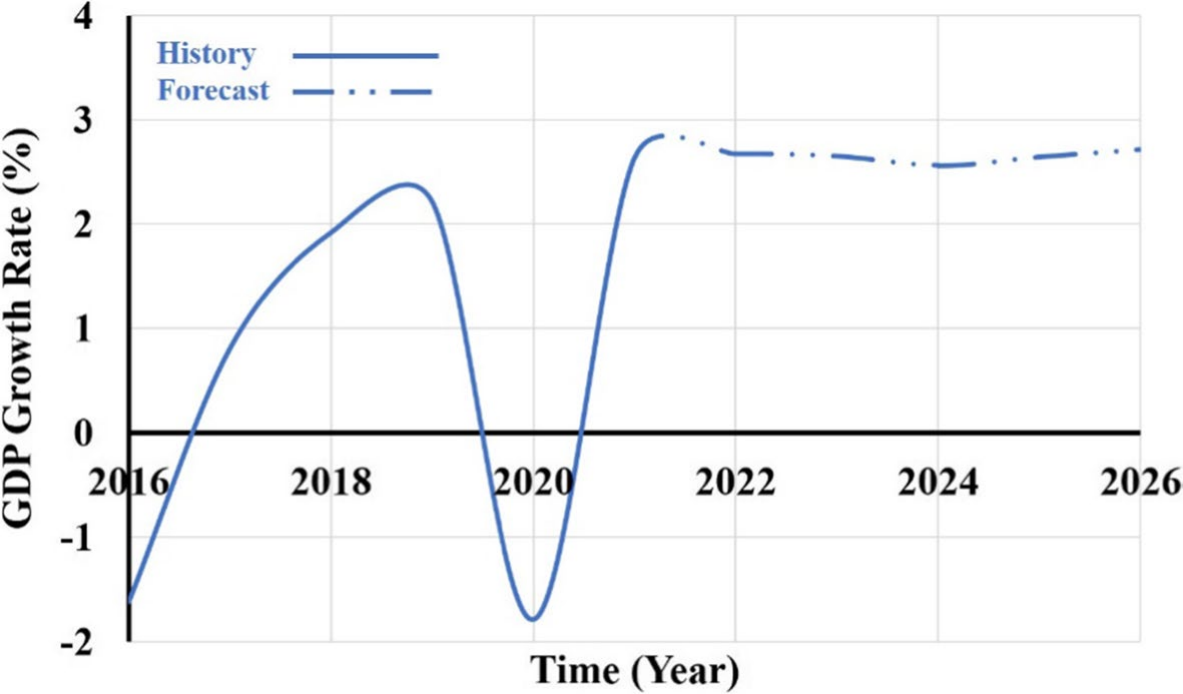
historical and forecasted GDP growth rate in Nigeria (IMF, 2021)

This ravaging economic recession is among the factors compelling several Nigerians to use technologies that are not environmentally friendly. The inadequate supply of electricity in the country is caused by corruption, government negligence, outdated infrastructures, illegal electricity connection and consumption, and so much more (Roy et al., 2020). The decline and instability of the GDP growth rate could influence the rating of the country's economy to high-risk rating; thereby, potential investors would be scared making considerable commitments in the region. Figure 4 illustrates the historical GDP growth and forecasted rate.

### Nigeria's energy crisis

The continuous rapid population increase and over-reliance on fossil fuels as sources of energy and the primary sources of revenue are causing several economic drawbacks in Nigeria. The increase requires an inevitable increase in energy supply to support livelihoods, industrialisation, increased food demand, and improved living standards. The country's electricity supply is inadequate and unstable and does not meet the demand. It can be easily understood that the country's rapid population growth is one of the problems that have necessitated a swift increase in energy demand in the country.

**Fig. 5:**
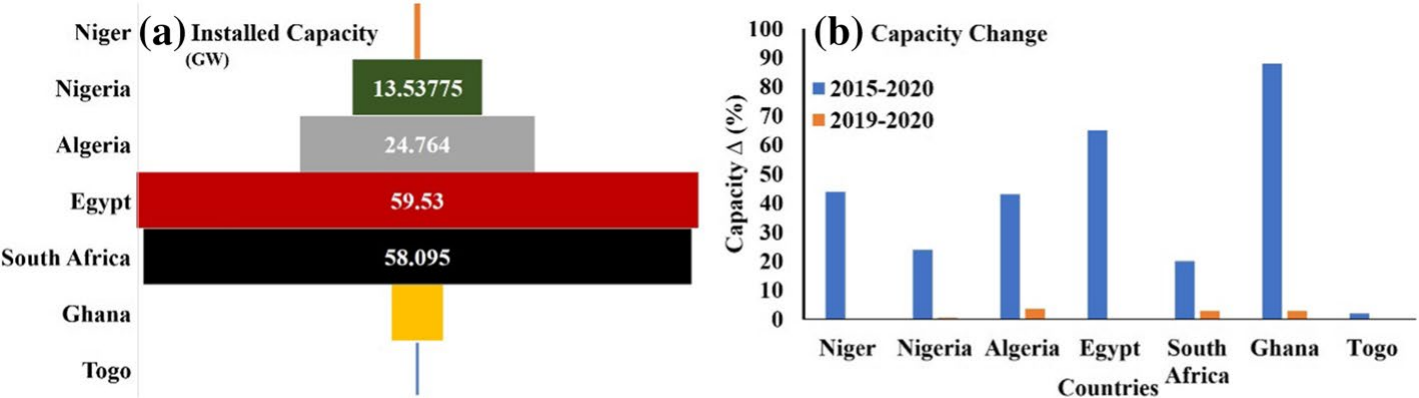
Variation of **a** installed capacity and **b** capacity change, including historical shifts (IRENA, 2021 (a))

Currently, Nigeria has an electricity installed capacity of 13.5 GW; however, compared to some developing countries in the same region, it is observed from Fig. 5a that capacity is minimal and requires significant addition. In addition, it is also observed from Fig. 5b that there is very minimal change in the country from 2019 to 2020 compared to the same countries having greater capacity. It shows that although the country's electricity installation capacity is low, there is still minimal improvement recorded in 2019–2020.

**Fig. 6:**
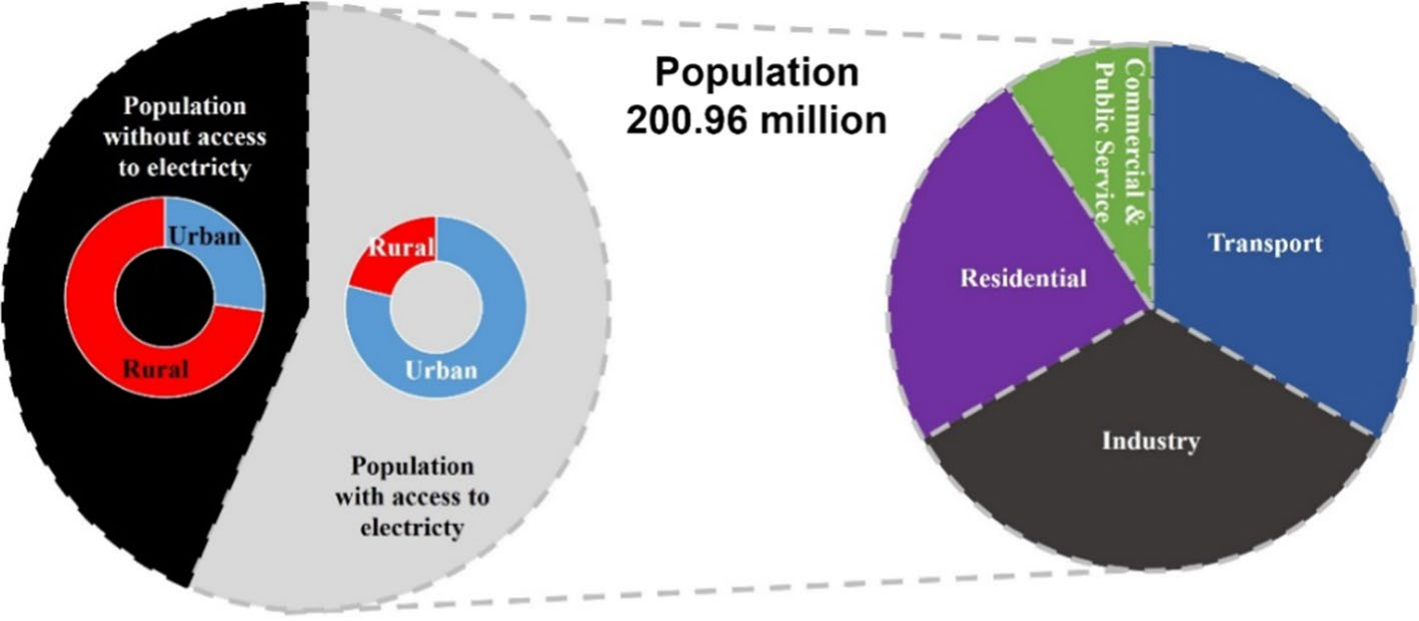
Energy consumption overview in Nigeria (World-Bank, 2021)

About 56.5% of Nigeria's population is connected to the national grid, receiving erratic and irregular electricity supply (World-Bank-Group, 2020). Therefore, about 45% (~ 90,000,000) do not have access to the national electricity grid, and about 80% of the population which lacks access to the national grid lives in rural communities (World-Bank-Group, 2020). The variations are further illustrated in Fig. 6, showing consumption according to the sector. According to Emetere et al. (2016), the few Nigerians connected to the national grid barely have 5 (Five) hours of constant and steady electricity supply daily. According to Oyedepo (2012b), 87,639 reported that the minimum system average interruption duration index (SAIDI) in Nigeria is high. In 2016, Nigerian electricity consumption per capita was about 0.14 MWh/Capita, and in 2018, it is still 0.2 MWh/Capita, which is very low compared to other developing countries (IEA, 2021a, 2021b, 2021c).

According to NERC (NERC, 2020), the peak generation was attained on 31 January 2019, where about 9 MW (201.340 GWh) was generated and supplied, which is a one-day event. It was not repeated as the generation fluctuates between 3 and 5 MW (NERC, 2020). This supply was achieved from various power generation companies, which include Power Holding Company of Nigeria (PHCN), Niger Delta Power Holding Company (NDPHC), Nigerian integrated power projects (NIPP), and other independent power plants (IPP). Other ongoing natural gases and hydropower projects are taking place around the country to increase the supply mix, but not all these would be adequate to satisfy the local demand.

The primary source of electricity in the country is thermal (powered by fossil fuel) and hydro. In 2018, Nigeria produced 36.277 TWh of electricity distributed in the following manner: 6.458 TWh of hydroelectricity, 0.028 TWh solar, and 29.791 Gas TWh, as illustrated in Fig. 1, which highlights the previous trend (IEA, 2021a, 2021b, 2021c). Electricity production from renewable sources, excluding hydroelectric, has started increasing, but the country is far from reaching its solar energy target, which is 500 MW by 2025, to provide 80% of its population with clean energy (IEA, 2021a, 2021b, 2021c). By 2030, which is the deadline for sustainable development goal, Nigeria's electricity consumption demand is forecasted to be estimated to reach; a peak demand of 24,481 MW, the annual demand of 115,466 GWh, the peak demand of 18,270 MW, and annual demand of 86,175 GWh (Adeoye and Spataru, 2020).

**Fig. 7:**
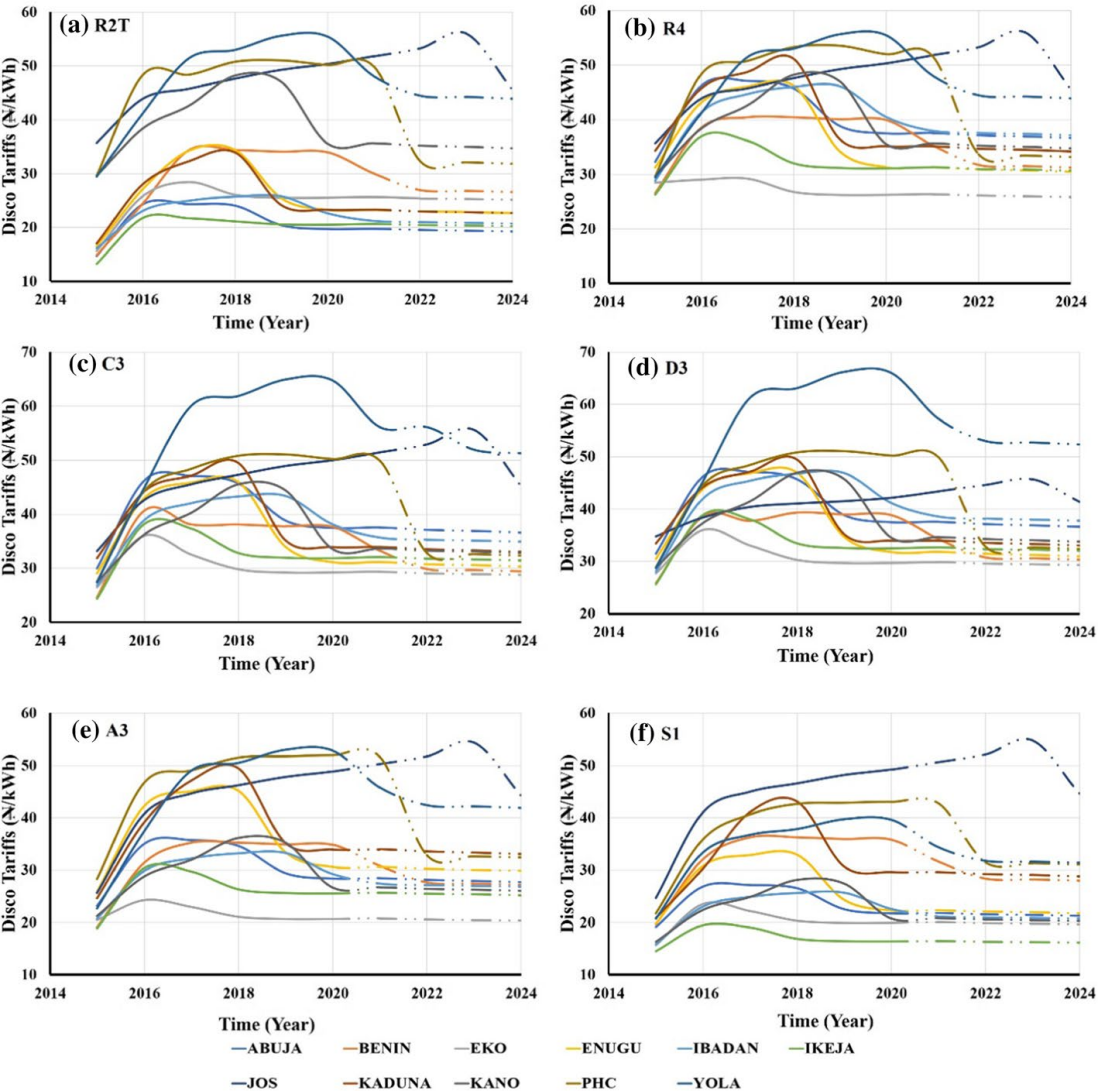
Tariff variation across the regions and DisCos in Nigeria with **a** R2T showing the residential three-phase tariffs; **b** R4 showing the unique residential of the individuals with their step-down transformers; **c** C3 showing the commercial sector tariffs; **d** showing the industrial sectors tariffs; **e** A3 special tariffs such government residents and establishments; **f** L1 showing street-lightening tariffs across the all the DisCos. The dotted lines represented the planned price to be implemented in the nearest future (NERC, 2021)

The values provided in Fig. 7 show that tariffs are significantly lower in the business capital Lagos (EKO and Ikeja) compared to the far Northern DisCos (Yola and Jos). This is assumed to be related to generation site proximity, terrain, and transmission requirements. Transmission infrastructure cannot be omitted as a factor. The industrial and commercial sectors show higher tariffs rates across various DisCos. However, it is observed that all tariffs will start declining, which is assumed to be in relation to the planned increase of renewable energy technology in the supply mix to boost generation capacity.

In Nigeria, about 70% of its population depends on firewood to generate energy, and about 80% rely on combustible biomass (Emodi et al., 2017; Mohammed et al., 2013). In 2013 more than 95% of rural dwellers and about 49% of urban dwellers used solid fuels for energy production (WHO, 2017). Based on these assertions Majority of Nigerians rely heavily on firewood. They are causing two significant negative impacts: emissions of harmful gases that cause air pollution and continuous deforestation. These have profound implications for both our ecosystem and its inhabitants. Nigerians are buying the generators regardless of the negative implications they might cause. In 2016, about 61,664.272 kt CO_2_ was emitted from liquid fuel consumption, about 121.011 kt CO_2_ was emitted from solid fuel consumption, and about 31,862.563 kt CO_2_ from gaseous fuel consumption (WorldBank, 2017c). These emissions are mainly from the industrial and residential application of fossil-fuelled generators to complement the energy supply, posing a severe negative consequence to humans and the environment.

Akinyele et al. (2017) stated that the continuous release and emission of anthropogenic pollution from fossil-fuelled generators contributes to climate change. It promotes GHG emissions (Green House Gases), detrimental to human health. Usman et al. (2015) documented a report where a family of seven people was found dead due to the fumes (by-products) of fossil-fuelled generators in Lagos. Similarly, Ayodele and Ogunjuyigbe (2015) reported that a family of 4 people was found dead in September 2014 because of diesel generators' by-products (carbon monoxide). They also reported that by-products of fossil-fuelled generators killed 4 (four) children and their 80 (eighty)-year-old grandmother. According to World Health Organisation (WHO) (WHO, 2016), about 95,300 deaths per year were recorded due to Nigeria's indoor air pollution. Emodi et al. (2017) also stated that about 79,000 Nigerians died due to indoor smoke inhalation caused by dependence on firewood. The by-product emissions from these generators are harmful gases and are very dangerous.

**Fig. 8:**
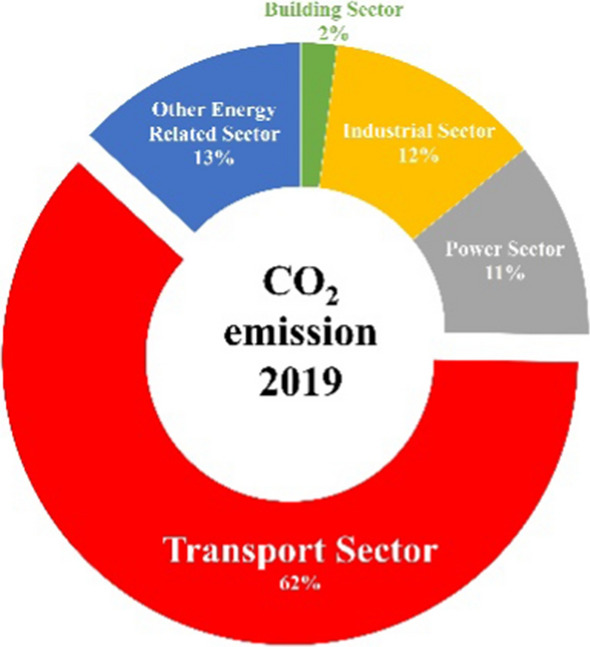
Variation of CO_2_-related emission from fossil fuel by sector (MtCO_2_/year) (Climate Transparency, 2020)

Impact of the oil and gas sector generates a large amount of pollution from oil spills to emission of greenhouse gases at both industrial level and residential levels, which exposes about 94% of the population to air pollution compared to 72%, which is the average rating for sub-Saharan Africa (Babatope & Solomon, 2020). In 2020, the pandemic reduced the global CO_2_ emission, and the air quality of Nigeria improved and aided the country in reducing annual emissions as planned under the Paris Agreement. The reduction is related to the shutdown of the transport sector activities, which is the major contributor to the countries CO_2_ emission (Climate Transparency, 2020). The distribution of CO_2_ is illustrated in Fig. 8.

**Fig. 9:**
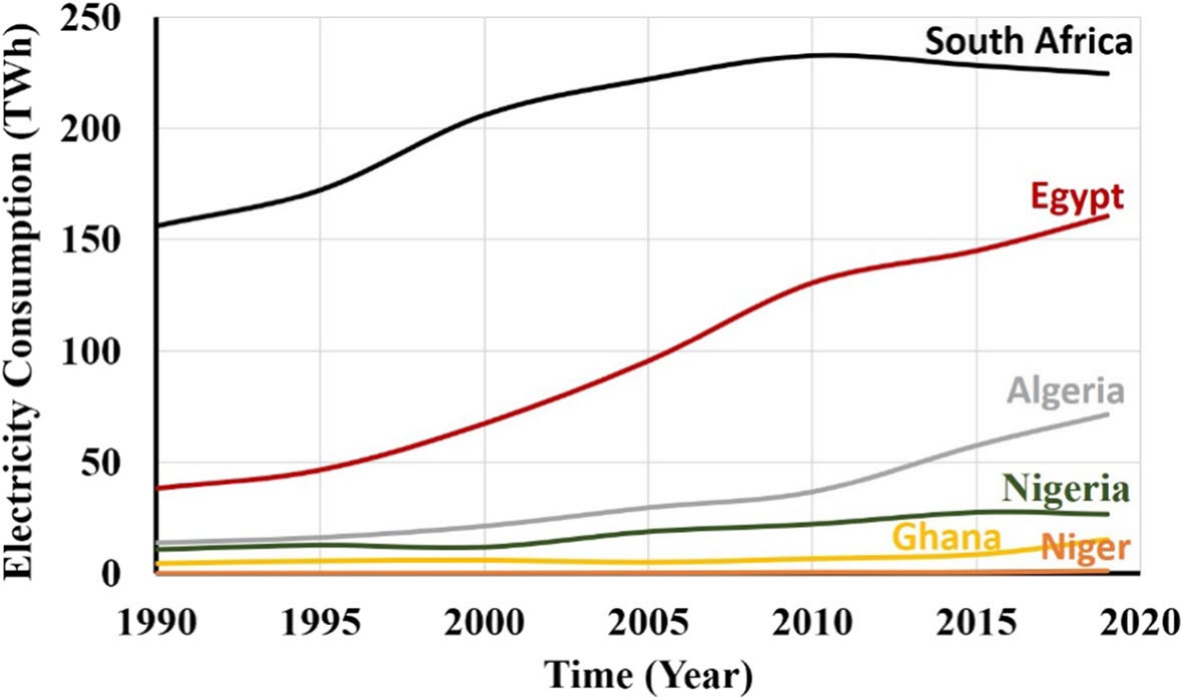
Variation of historical electricity consumption across sub-Saharan Africa (IEA, 2021a, 2021b, 2021c (a))

Electricity consumption by the industrial sector of the national grid has been declining but increasing domestic use. According to Oseni (2011), industrial energy consumption decreased from 62.9% to 9.7% for 35 years. Furthermore, about a 20% reduction was recorded in 2011 alone. Also, the researcher further stated that domestic electricity consumption increased from 37.1% to 63.8%. This shows that the population is increasing, and industries are either shutting down and/or using alternative sources to generate their electricity. The continuous multiple power outages and unpredictable supply have an extremely negative impact on Nigeria's economy. It has paralysed the country's industrial sector by compelling them to adopt self-energy generation using fossil fuel generators, increasing the production cost. It is noteworthy that Nigeria's electricity consumption is very low, according to Fig. 9. If related to the nation's population, it should signal lower supply and low electricity accessibility, leading to economic instability and less development.

**Fig. 10:**
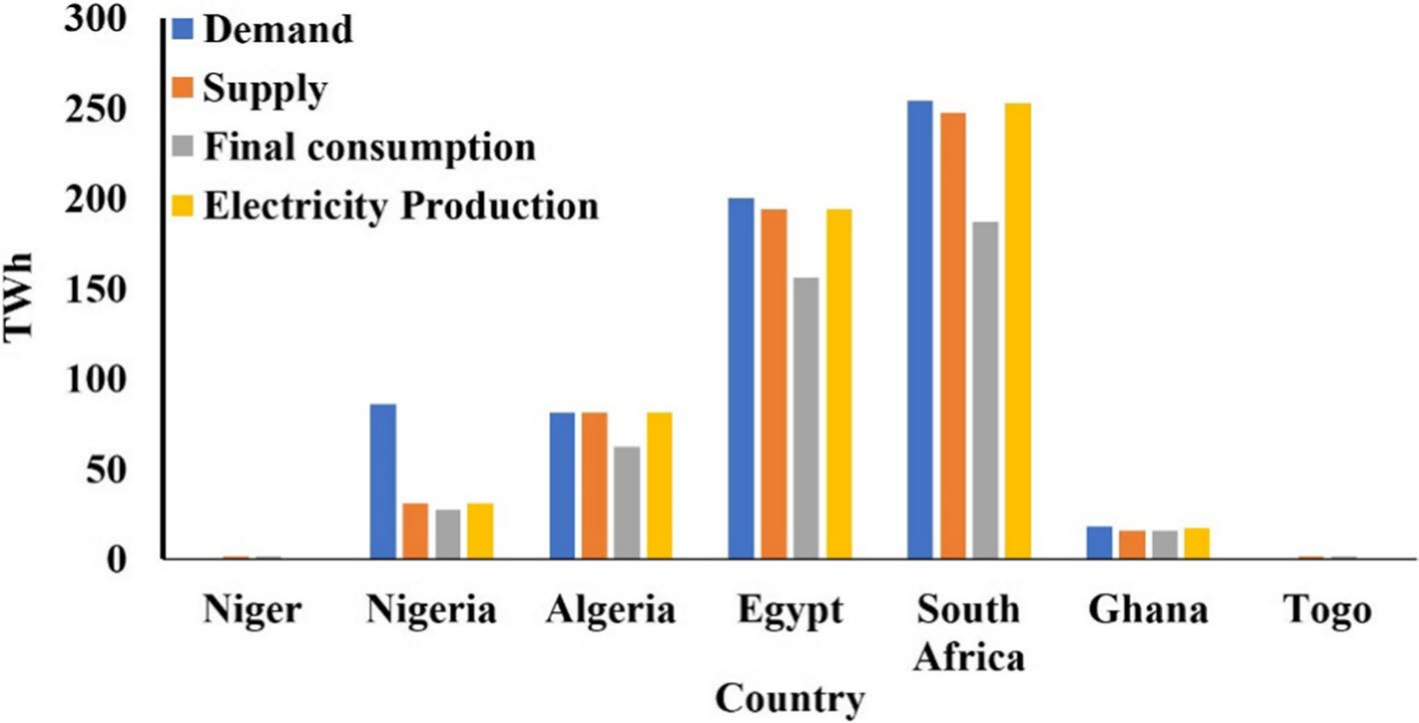
Variation of electricity production, demand, supply, and final consumption across the sub-Saharan African region (IEA, 2021a, 2021b, 2021c (a))

Under a multination agreement, Nigeria generated electricity and agreed to sell it to neighbouring countries, even if the supply was insufficient to meet its local demand. This created a wide gap between demand and supply. Figure 10 shows that electricity demand is high, supply is low, and the generation is while final consumption is even lower than what is produced. Compared with other developing nations in the region, such as Algeria, there is no gap between demand and supply, while Egypt and South Africa have a minute gap.

**Fig. 11:**
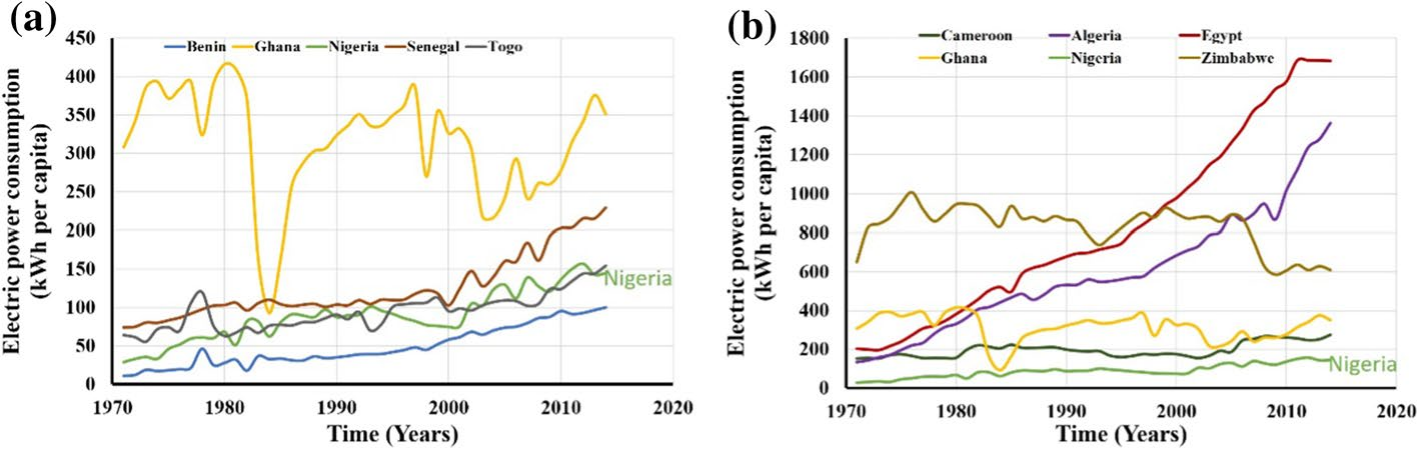
The electricity power consumption in kWh per capita with **a** highlighting variation between countries in West Africa and **b** showing a variation of consumption between countries across the African continent (IEA, 2021a, 2021b, 2021c)

Figure 11a shows that Nigeria's energy consumption per capita is low compared to Ghana and Senegal, and it has almost a similar value to Togo. Compared with some developing countries across the sub-Saharan African region in Fig. 11b, it was observed that the country is recording very minimal improvement over two (1980–2000) decades, and some slight increase is recorded in the last two decades.

Adaramola et al. (2014) stated that a recent survey shows that the self-generation of electricity in producing an item in Nigeria is about 30–40% of the production cost, while in countries like China, it is just 5% to 10%. According to AfDB (2017), Nigeria's manufacturing sector has recorded a general decline in 2016. It has been estimated that about 272 firms have shut down, which causes industrial capacity utilisation of electricity to automatically drop to 35.4% in 2016 from 51.4% in 2015 (AfDB, 2017). Similarly, Aliyu et al. (2015) stated that to produce an item in Nigeria will cost nine times (9X) higher than to produce it in China because self-energy generation constitutes about 40% of the total production cost. The researchers further stated that MAN (Manufacturers Association of Nigeria) reported that about USD 11,340 million is spent on fuelling and maintaining fossil-fuelled generators weekly. These data indicate that the lack of a stable electricity supply seriously affects the country's industrial and manufacturing sectors. Worldbank (2021) reported that losses related to the unreliable electricity supply are estimated to be around USD 26.2 billion (₦10.1 trillion).

The high cost of production will increase the retail cost of an item, and if an alternative is available, the consumer might want to obtain the cheaper option. Self-energy generation is not an alternative to enterprises such as communication companies in Nigeria because it is almost their primary source of electricity. These companies require a steady and interrupted electricity supply to power the network and support communication technologies.

Consumption of electricity has significantly reduced in the industrial sector but increases in residences. According to Oseni (2011), energy consumption decreased from 62.9% to 9.7% from 1970 to 2005 and reduced by 20% in 2011. Similarly, the researcher further stated that electricity consumption increased from 37.1% to 63.8% in residences in the same period.

Apart from the adverse effects of fossil fuel generators, their maintenance cost is very high. The Nigerian government is losing huge sums due to power outages. Trillions of Naira (Nigerian Currency (₦)) are spent running and maintaining fossil-fuelled generators. The Nigeria Council for Renewable Energy (CREN) estimates that power outages lead to a loss of ₦126 billion (USD 984.38 million) annually (CREN 2009, cited Nnaji et al. (2010). In Nigeria, most commercial businesses, either large or small, rely on generators as their source of electricity. According to Oyedepo (2012b), the South African telecommunication giant and the largest mobile company in Nigeria (MTN) is estimated to have 6000 generators installed in their various base stations in the country and are operating for about 19 h/day, which requires diesel that totals about USD 5.5 million monthly. Similarly, it applies to their other counterparts, such as Airtel, GLO., and Etisalat (Yusuf, 2014).

Akuru et al. (2017) reported that about ₦ 350 billion is spent on an annual basis by industries on fuel to power generators to produce electricity, and this is part of the estimated total of ₦796.4 billion spent in the country on an annual basis on fuel generator sets for electricity generation. Similarly, Ayodele and Ogunjuyigbe (2015) also reported that it had been estimated that ₦3.5 Trillion had been spent on running and maintaining fossil-fuelled generators in Nigeria. This has placed Nigeria as the highest user of generators globally, with more than 60 million households having their generators, making the country the highest importer of generators in the world (Ayodele & Ogunjuyigbe, 2015). Considering the high costs of fossil fuel alone used in running power generators in Nigeria, it is evident that renewable energy such as solar energy systems is becoming cheaper alternatives (Mohammed et al., 2017).

According to Oyedepo (2012b), despite the abundant natural resources reserves (fossil fuel) in Nigeria, which are underexploited, with the present rate of exploitation and extraction, it has been estimated that reserves (of non-renewable energy) will be low to the point where it would be uneconomical to continue the exploration in the next 40 years. It will, therefore, become necessary to start exploring and triggering the adoption of other alternative sources such as renewable energy systems and implement them as soon as possible. The renewable energy potential in the country is enormous, and according to Sambo (2009), renewable energy in Nigeria is a viable solution to the current energy crisis in the country. The country's potential is more than 1.5 times fossil fuel resources in energy terms (Shaaban & Petinrin, 2014). Harnessing renewable energy resources such as solar energy can solve all the persisting problems affecting the country's electricity supply, thereby promoting sustainable urban and rural socio-economic development. Aliyu et al. (2017); Shaaban and Petinrin (2014) point out that solar energy is the backbone and driving force of other renewable energies such as hydro, biomass, and wind. Ozoegwu et al. (2017) also believe that solar energy should be considered the highest priority energy source in Nigeria and worldwide.

## Nigeria's energy outlook

This section briefly discussed the fossil fuel resource of the country and the electricity infrastructure. Fossil fuel potential is provided to highlight why the country is using such resources to generate electricity, and the electricity infrastructure subsection is to demonstrate the capacity level of the installation across the country. This information could provide insight to the reader and future writers on the gaps and recommend an appropriate solution.

### Nigeria's oil and gas

Nigeria is endowed with abundant natural resources such as fossil fuels (oil and gas). Based on these resources, population, and other factors, the country is categorised as the giant of Africa. The country relies on the oil and gas industry as its primary revenue source (Adom & Adams, 2017). Unfortunately, these resources are not correctly used to resolve the persisting socio-economic problems and other challenges, including the country's energy crisis for more than two decades. Substantial financial resources of over ₦2.1 trillion from 2014 to 2020 obtained through oil and gas exploitation were invested by the government through its central bank (Anyaogu, 2021) towards boosting the electricity sector development; however, several factors such as policy instability and corruption hindered the improvement of the sector (Roy et al., 2020).

Nigeria is a member of the Organisation of the Petroleum Exporting Country (OPEC). When combined, Nigeria and Libya's crude oil reserve accounts for 2/3 (two-thirds) of the African oil reserve. In Nigeria, oil was first discovered in a town called Oloibiri, in Bayelsa State, in the Niger Delta, South-South region, in 1956, just before independence. The country has witnessed a steady increase in exploration activities over the decades. This exploration growth arises from a funding scheme that has to do with production-sharing arrangements. The exploration activities are onshore and offshore (in the Atlantic oceans). In 2015, the country had about 29 active oil rigs, a pipeline network of 5001 km around the country, with about 22 (twenty-two) oil depots (Anumaka, 2012; OPEC, 2021). According to the Department of Petroleum Resources (DPR) (DPR, 2021), Nigeria has five functioning oil and gas refineries (Port Harcourt Refining Company (PHRC) in New Port Harcourt, WRPC in Warri, Kaduna Refining and Petrochemical Company (KRPC) Kaduna, PHRC Old Port Harcourt, and Niger Delta Petroleum Resources (NDPR) with a total production capacity of 446.0 (1000 barrels per day (b/d)) but produces only 8.2 (1000 b/d), while the country's oil demand is about 469.8 (1000 b/d) (OPEC, 2021). These clearly show the low performance of local refining capacity, as crude oil is exported for refining purposes and then imported back for local consumption. This low performance is caused due to poor maintenance, inadequately trained professionals, underfunding, corruption, and vandalism (Roy et al., 2020; Otombosoba, 2021).

Nigeria has enormous oil resources, yet its populace frequently suffers from shortages of oil products. Based on the above facts relating to insufficient refining capacity, the country's crude oil export reached about 2008.2 (1000 b/d) (OPEC, 2021). The country has a proven crude oil reserve of 36,890 million barrels (OPEC, 2021). In 2015, Nigeria produced about 2329 b/d, while in 2016, the country produced about 2053 b/d, a decrease of about − 11.9%, and a further decline to 1737.4 barrels/day in 2020 (OPEC, 2021). Its refining capacity is about 446.0 (1000 b/d), and the output of the refined petroleum products is about 28.3 (1000 b/d), while the country's oil demand is about 469.8 (1000 b/d) (OPEC, 2021). In 1999, it was documented that the country had a proven reserve of 25 billion barrels. In 2004, it increased to about 34 billion barrels in 2017, and it would continue to increase to about 37 billion barrels, and by 2030 it is projected to reach 68 billion barrels (Anumaka, 2012) potentially. In 2015, Nigerian OPEC production percentages were reduced to 5.8% compared to 7%, which had a devastating effect on the country's economy because of the overdependence on the oil and gas sector (Wijeratne et al., 2016).

The gas reserves are proven to be extremely large at 3 (three) times the amount of oil in the country, making the second global reserve after Algeria. In 2020, Nigeria had a proven natural gas reserve of about 5.4 trillion m^3^ (cubic metre), equivalent to 190.4 trillion ft^3^ (cubic feet). In 2019, the country produced 49.3 billion m^3^, which is 45.1 tonnes of oil equivalent of natural gas, while in 2016, it produced 48.3 billion m^3^ (BP, 2020; OPEC, 2021). About 35.9 billion m^3^ of natural gas was exported (OPEC, 2021). Nigeria had been wasting its gas through gas flaring in the Niger Delta until 1990 when the Nigerian National Petroleum Corporation (NNPC) concluded the agreements and established Nigeria LNG (Liquefied Natural Gas) 2 (two) years later, and commenced production in 1999 (Anumaka, 2012). According to Shaaban and Petinrin (2014), about 45.8 billion kWh of heat discharge into the South-South region of Nigeria from gas flaring of about 1.8 billion ft^3^ of gas daily led to the country being rated among one of the highest CO_2_ (carbon dioxide) and GHG (green house gas) producers in the world. According to Anumaka (2012); Shaaban and Petinrin (2014), Nigeria is assumed to be one of the world's highest gas flaring nations. Anumaka (2012) further stated that the country is losing vast quantities through this gas flaring, with an estimated USD 18.2 million every day. These vast resources could generate electricity and other developmental and infrastructural projects.

Coal is the oldest natural resource discovered and exploited in Nigeria. It was discovered in 1909, and mining began years later, having a recorded production output of 24.500 tons in 1916 (Anumaka, 2012). About 95% of the production was consumed locally for transportation (railways), industrial activities (cement production), and electricity production, unlike oil and gas. The country has a proven reserve of about 639 million tonnes of coal, and the inferred reserves are estimated at 2.75 billion tonnes (Anumaka, 2012). The utilisation of coal drastically dropped just after the discovery of crude oil. The diesel-powered trains and various sources of electricity generation were also introduced in the country. The available potential can still be utilised in production activities and power generation, but its by-products severely negatively affect human health and the environment.

## Nigeria's renewable energy outlook

Renewable energy is the energy that is replenishing and non-deplete-able. It could guarantee energy security, promote national economic development, reduce/eliminate GHG (Green House Gas) emissions, conserve non-renewable energy, and eliminate or reduce their cost price (Okoye et al., 2016). The country has enormous potential for renewable energies, which are primarily unutilised. As provided in the subsequent section of this report and Table 1, the literature has established that Nigeria has abundant renewable resources such as biomass, hydro, solar, and wind energy. The potential of these renewable energies is discussed in the subsequent section, and a summary is provided in Table 1.

**Table 1:** Summary of renewable energy potential in Nigeria (Alabi et al. 2018; Giwa et al., 2017; GSA, 2020; and Brimmo et al. 2017)

Resources		Potential capacity	Utilisation
**Biomass** (Giwa et al, 2017)	Agricultural Resources	697.15 TJ	43.4 million tonnes of firewood/yr
	Municipal Waste	2.04 million m^3^	
	Animal Waste	6.8 million m^3^	
	Forest Resources	73,800 m^3^	
**Hydro** (Brimmo et al. 2017)	Large Scale	11,250 MW	1972 MW
	Small Scale	3,500 MW	64.2 MW
**Solar** (GSA, 2020)	Northern Part	25.2 MJ/m^2^/day (9 h)	About 28 MW solar PV standalone across the country. No solar thermal electricity
	Southern Part	12.6 MJ/m^2^/day (3.5 h)	
**Wind** (Alabi, 2018)	Northern Part	4.0–5.12 m/s @ height of 10 m	2 × 2.5 kW electricity generator; 10 MW wind farm in Katsina (Still under construction)
	Southern Part	1.4 to 3.0 m/s @ height of 10 m	

### Biomass

Biomass is the non-fossil organic materials or any living matter derivable from sources such as plants and animals, including their by-products that can be used to generate or form useful energy (Akuru et al., 2017). Biomass is a chemical compound containing free energy, which can be in solid, liquid, and gaseous form (Aliyu et al., 2017). Electrical and mechanical energy can be generated when energy breaks down the compound's molecule, generating heat. Nigeria possesses abundant Biomass resources, including but not limited to fuelwood, charcoal, shrubs, grasses, sawdust, forestry waste, crops, animal waste, aquatic wastes, municipal waste, and industrial waste (Akuru et al., 2017; Shaaban & Petinrin, 2014). These are categorised into agricultural, municipal solid waste, forestry resources, and animal waste (Akuru et al., 2017; Aliyu et al., 2017; Mas’ud et al., 2015; Shaaban & Petinrin, 2014). It is noteworthy that the most utilised biomass in Nigeria is firewood (fuelwood). Monyei et al. (2017) stated that about 90% of households in Northern Nigeria use firewood for cooking and further estimated that about 72% of the population depend on fuelwood for cooking daily. Emodi et al. (2017) also supported this claim stating that this is also the case for about 70% of the country's population. Shaaban and Petinrin (2014) reported that Nigeria has an estimated capacity of about 8 × 10^2^ MJ of biomass resources, 13 million hectares of woody lands, and 61 million tons per annum of animal waste crop residue. The potential of the various biomass sources mentioned above is discussed in the paper's subsequent section.

The agricultural resource is categorised as either food crops or agricultural residue, and these resources are currently utilised for domestic cooking or industrial application. The various agricultural resources available in Nigeria include cassava, sugar cane, cornstalk, rice husk, palm kernel shell, coconut, cashew nuts, groundnut, sesame, jatropha curcas, and castor oil. These resources have excellent potential for producing biofuel. Due to overdependence on conventional fossil fuel, the country has very few biofuel companies. It has been reported that Nigeria's agricultural resources potential is estimated as 697.15 TJ (Giwa et al., 2017; Mohammed et al., 2013; Sambo, 2009). This is a considerable amount and can be viable for useful energy generation.

Municipal solid waste resources are substances produced by human activities. Due to Nigeria's high population, the country has enormous potential municipal solid waste resources, with an average per capita municipal solid waste varying between Nigeria's cities. According to Giwa et al. (2017), the country generates about 74,428.85 tons of municipal solid waste per day, producing 2.04 million m^3^. The municipal solid waste can be converted to bioenergy through anaerobic digestion, steam boilers, resource burning, and gasification. A small number of private and government companies generate adequate energy amounts in the country from this source.

Animal residue implies animal waste products, dumps, and remains. This chemical compound can generate energy using various methods and is a very efficient renewable energy source. Nigeria access these resources through animal dumps from cattle, poultry (chicken, turkey, and other domestic birds), goat, sheep, pigs, camels, horses, donkeys, and abattoir remains (consisting of blood, intestines, and other animal remains). Giwa et al. (2017) estimated that about 227,500 tonnes of animal waste is being produced daily, mostly in Northern Nigeria, where energy poverty is exceptionally high. This amount of waste can be converted to about 6.8 million m^3^ of biogas. This resource is not adequately utilised, with only a few companies using biodigesters were being identified in the country (Giwa et al., 2017; Mohammed et al., 2013; Sambo, 2009).

As earlier stated, firewood is the most used and most widely available biomass resource in Nigeria. The country has a large landmass, and it has been reported that 12% of the land is mainly forest (Mohammed et al., 2013). In 2014, Nigeria had an estimated agricultural area of 70,800,000 ha and a forested landmass of 7,402,600 ha (FOA, 2018). The country's firewood potential was estimated to be about 73,800 m^3^, and the firewood used in the country is approximated at about 6 billion MJ in energy content (Giwa et al., 2017). Overdependence on firewood is continuously increasing due to the continuous increase of population economic problems, and this has already led to excessive deforestation and caused the loss of about 300 000 ha landmass (Giwa et al., 2017). The use of forest resources such as firewood promotes deforestation and gas emissions, detrimental to human health and the environment.

### Hydro

Hydropower energy generation is a process whereby energy is released to generate electricity using water body movement to rotate mechanical turbines (Ikem et al., 2016). The amount of energy generated depends on the volume and falling rate of the water. Hydro energy is the first and only renewable energy contributing to Nigeria's national utility grid's existing electricity energy supply mix.

**Fig. 12:**
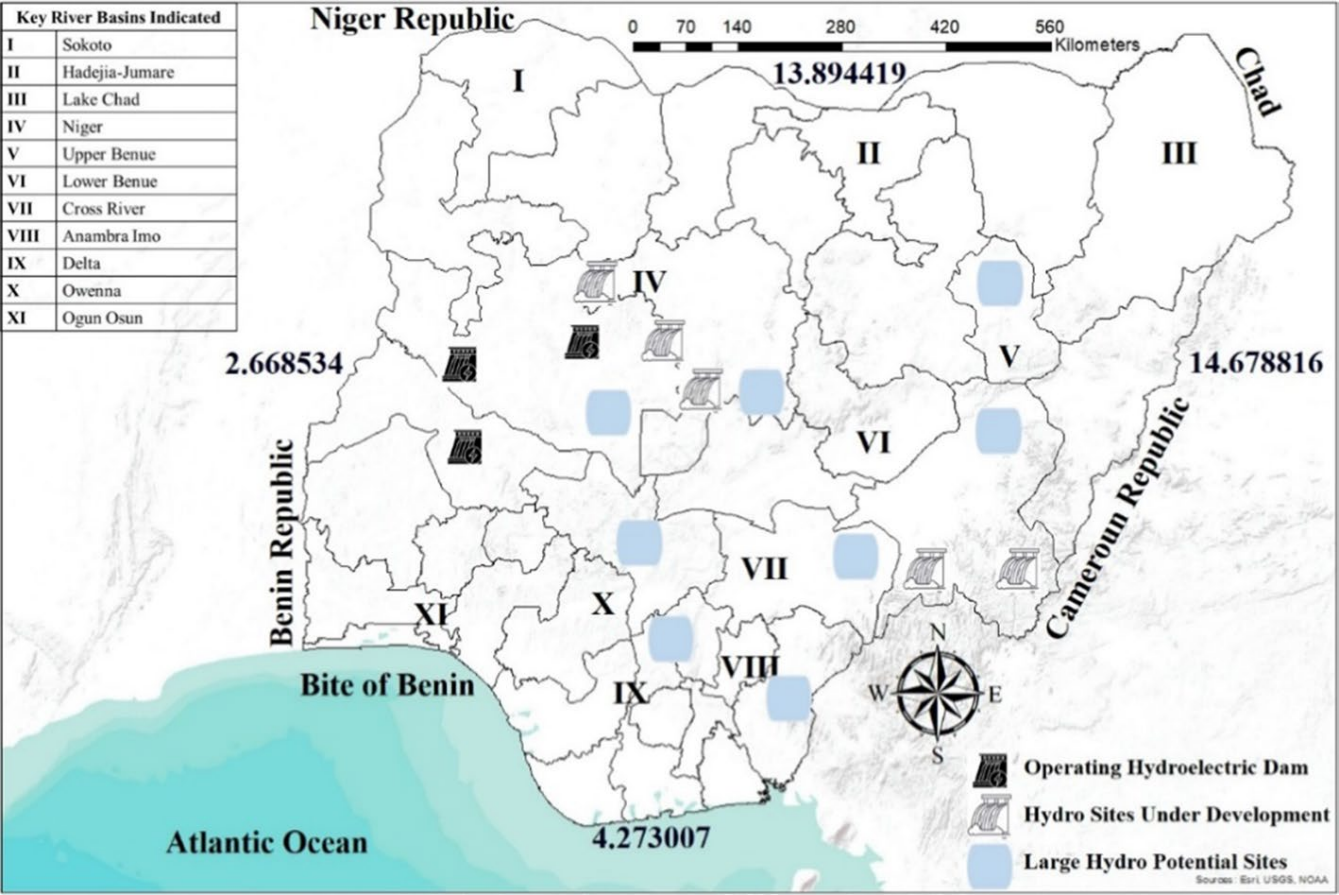
Hydroelectricity capacity including operational, under construction, and enormous potential sites (NBET, 2010)

Nigeria has two major rivers (river Benue and Niger) crossing the country, providing a total exploitable potential of about 14,750 MW. This translates to large hydro plants having the exploitable potential of about 11,250 MW, while the small hydro plants have 3500 MW from 286 sites consisting of 86 small sites, 126 mini dams, and 70 microdams across the country (Ikem et al., 2016; Vincent & Yusuf, 2014). Currently, it has an installed capacity of 1938 MW spread among three-generation plants Jebba Hydro Electric Plant (HEP) (578 MW), Kainji HEP (760 MW), and Shiroro HEP (600 MW) (Monyei et al., 2017). It has been established that Nigeria is an African country with the ninth highest economic potential for hydropower. Several ongoing hydro projects in Nigeria are supported by the World Bank, African Development Bank, UNIDO (United Nations Industrial Development Organization), and the Chinese Government to boost agriculture and generate electricity. Figure 12 shows the sizeable hydro plant sites and the rivers across the country.

### Wind

The wind is the movement of air masses triggered by differential heating of the earth's surface from the sun and its consequential pressure disparities. Wind energy is generated when kinetic energy is exploited. It causes the turbines' shaft to rotate (mechanical energy), thereby generating electricity (Akuru et al., 2017). Wind speed is the most crucial factor in wind energy, and the available wind speed in Nigeria varies with season and location. Many researchers have investigated the average wind speed in Nigeria, and most of them concluded that the available wind speed in the Northern part of the country is viable for electricity generation.

Ohunakin (2010) researched the wind and its characteristics and reported that Nigeria has an annual average wind speed of 4.570 m/s in the Northern region and 2.747 m/s in the Southern region height of 10 m. Similarly, researchers (Ikem et al., 2016; Sambo, 2009; Vincent & Yusuf, 2014) also report the wind potential, with an annual mean wind speed of about 4.0 m/s in the far North and about 2.0 m/s in the Southern coastal region at the height of 10 m. Akuru et al. (2017) also reported that the country has an annual mean wind speed of 2 m/s in the Southern coastal region and 8 m/s in the far Northern region at the height of 50 m.

**Fig. 13:**
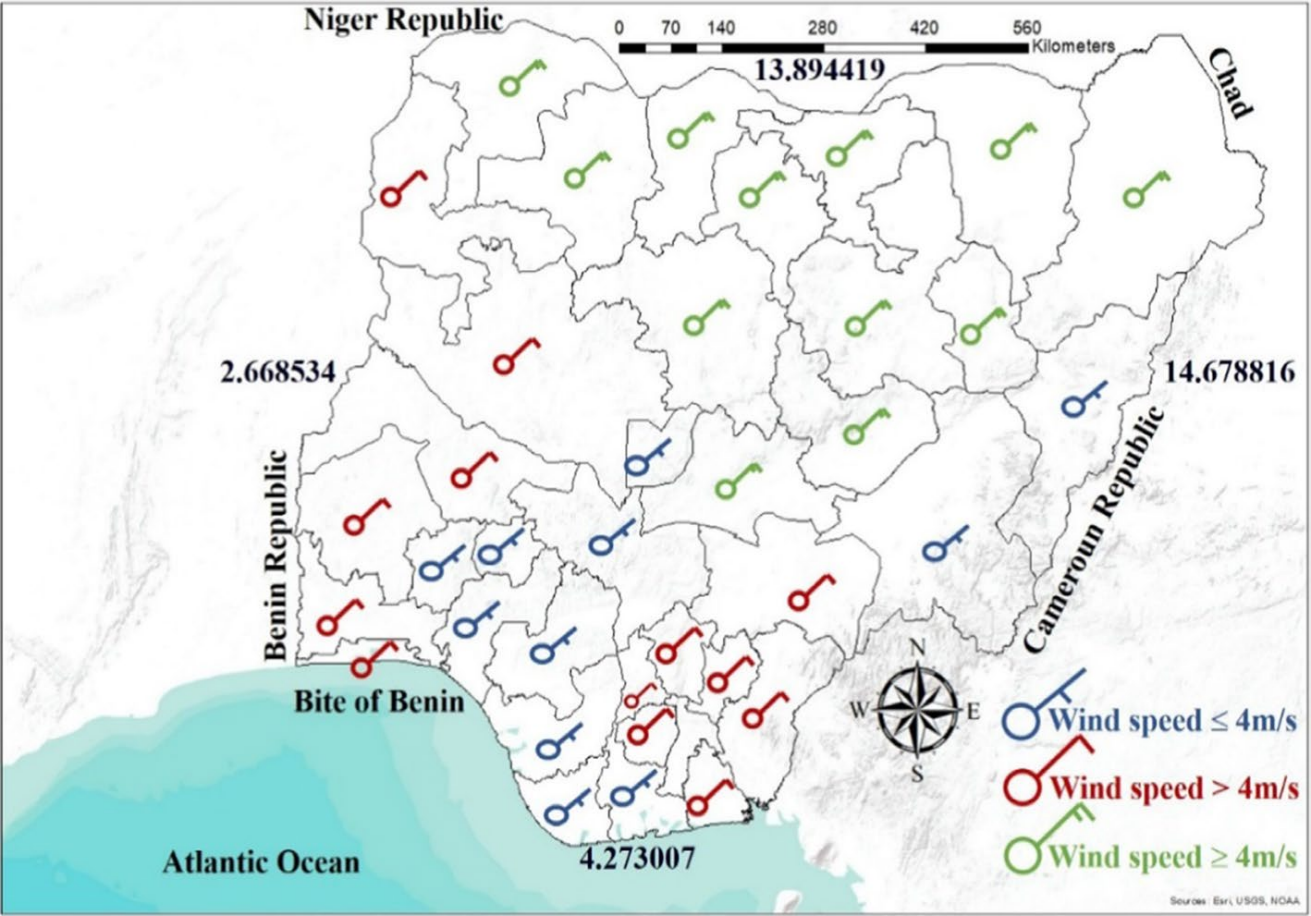
Wind potential with velocity variation across Nigeria

On the contrary, researchers (Mas’ud et al., 2015; Shaaban & Petinrin, 2014) reported slightly higher figures stating that Nigeria's wind speed ranges from about 1.4 to 3.0 m/s in the South to about 4.0 to 5.12 m/s in the far Northern region. Figure 13 shows the average wind speed of various locations in the country, and it is evident that the Northern part of the country has exploitable wind potential. According to the ECN, cited by Mas’ud et al. (2015), Shaaban and Petinrin (2014), Nigeria has a wind energy reserve at the height of 10 m that varies from as high as about 97 MWh/yr in Sokoto (a north-western state) and about 51 MWh/yr in gas (a north-central state) to about 8 MWh/yr in Yola (a north-eastern state). This figure highlights the potential of wind in Nigeria.

Despite this abundant wind energy potential in the Northern part of the country, wind energy does not contribute to the national supply mix. Few wind projects were recorded in the country, and the first and only sizeable notable wind farm project (10 MW project in Rimi village, Katsina State) that was meant to contribute to the national grid has been abandoned. The country's only successful, famous wind energy application is 0.75 KW in Dan Jawa Village of Sokoto State and 5KW power generating station in Sayya Gidan Gada, Sokoto State (Akuru et al., 2017; Mohammed et al., 2017). Another unique method of wind energy utilisation in the country is water pumping installed in the Kano state during the colonial era.

### Solar Energy

**Fig. 14:**
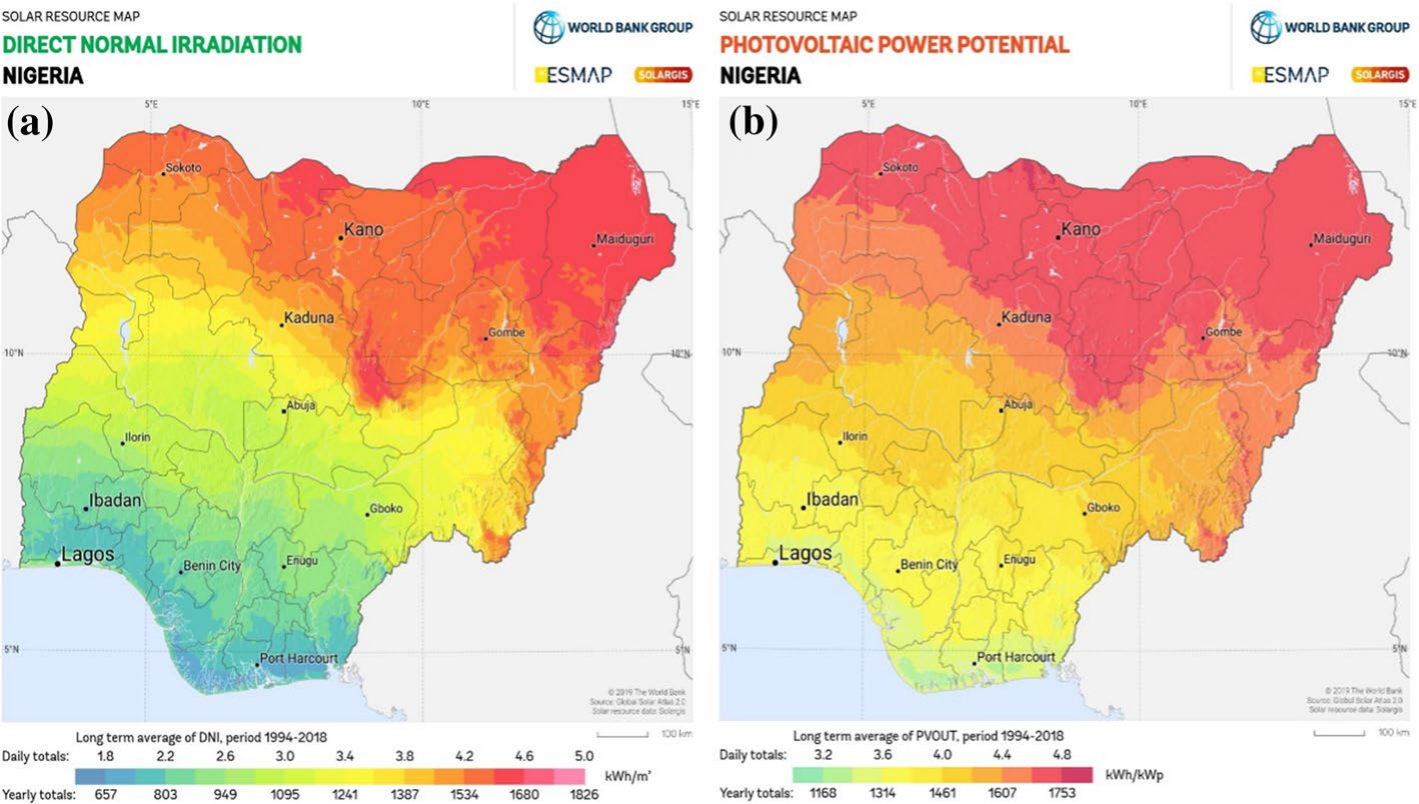
Direct normal irradiation and PV power potential (GSA, 2021)

Solar energy relies on nuclear fusion that emits from the sun's core. Nigeria is situated in the equatorial region, which exposes it to too much solar radiation. According to reports, Nigeria is endowed with intensive sunshine, with an average of 6.25 h per day, ranging between 9.0 h in the far Northern boundary and about 3.5 h in the coastal areas, meaning that Nigeria receives average solar radiation of about 12.6 MJ/m^2^ /day at the Southern coastal latitudes and about 25.2 MJ/m^2^ /day in the far Northern part of the country, giving the mathematical average as 18.9 MJ/m^2^ /day (Gaglia et al., 2017; Ilenikhena & Ezemonye, 2010; Nnaji et al., 2010; Ohunakin, 2010). This translates to an equivalent of 229.1667 W/m^2^ in power terms. Global Solar Atlas (GSA) (2021) provided the details of direct normal irradiation across Nigeria, with an average of about 724 kWh/m^2^ in the far Southern part and 1653 Wh/m^2^ in the far Northern region. This translates into a PV power potential of 1248 kWh/kWp in the South and 1756 kWh/kWp, and this information is further illustrated in Fig. 14.

With a total land area of 923,786 km^2^, Nigeria typically receives an impact of solar radiation of about 1500 × 109 MWh annually, with an annual average of 19 MJ m^−2^ day^−1^ (Ohunakin, 2010). An average of 6, 372, 613 PJ year^−1^ (approximately 1770 × 103 TWh year^−1^) of solar energy received in the entire land area is 120,000 times the total of PHCN (Power Holding Company of Nigeria) electricity generating capacity of 2002, and this energy value was estimated at 115,000 times the electrical energy generated by PHCN and about 27 times the value of total fossil fuel resources in the country (Shaaban & Petinrin, 2014).

## Solar energy technologies

Solar energy is inexhaustible and less pollutant, serving as a promising potential source for green electricity generation. Despite this massive amount of solar energy striking the earth's surface, several developing countries such as Nigeria encounter difficulties harnessing the resources. According to Mohammed (2017), several developed countries have recorded electricity stability after investing in solar. Investing in solar energy harvesting technologies can reduce or eradicate energy poverty in the developing world and promote the reduction of green house gases (GHG).

There are three significant ways to convert solar energy into electricity: solar photovoltaic conversion, solar thermal conversion, and concentrated solar power conversion. These technologies are devices that convert solar energy into a usable form of energy. These technologies are briefly discussed in the subsequent section of this paper, emphasising photovoltaic because it is the most utilised solar technology in Nigeria and worldwide.

Solar thermal conversion is achieved by using the Sun to heat a fluid to a certain steam level that can drive turbines to generate electricity. A solar thermal collector uses solar radiation to heat the fluid; then, the evacuating air is the steam that drives the turbines, and the heated fluid is transferred into a storage tank (Leggett, 2009). The required temperature for solar thermal depends on the intended application and the type of solar collector. Solar thermal has many other conversion techniques and can be used for various applications. In Nigeria, solar thermal is used to generate solar cooking, crop drying, incubating, water heating for industries, hospitals, prisons, and households (Aliyu et al., 2017). According to Renewable Energy Policy Network for the 21st Century (REN21) (REN21, 2020), solar water heating collectors had a cumulative global installed capacity of about 479 GW^th^ at the end of 2019, from a 1% decrease from the previous.

Concentrated Solar Power technology converts sunlight into energy using heat. It is designed to collect and concentrate sunlight to generate heat that drives energy generators such as turbines or solar cells to produce electricity (Green-Rhino-Energy, 2016). Devices such as lenses and mirrors are used to collect, focus, and concentrate solar radiation onto the photovoltaic cell to increase the device's generation and efficiency (Shanks et al., 2016). This form of electricity generation requires immediate utilisation and a cooling mechanism to protect from overheating in the system as the temperature is excited in a split second (Siyabi et al., 2017). It has been developed to reduce the photovoltaic system's cost by illuminating solar cells with a very high-intensity light using the lenses.

There are various methods in which concentrated solar energy generation can be accomplished, either trapping the light or utilising the refractive and reflective devices to increase sunshine intensity. The technology is in the market's introductory stage, and in 2019 it has an installed capacity of about 6.2 GW (IRENA, 2018). There is no record of the concentrated solar power technology installation in Nigeria.

### Solar photovoltaic

This device uses photovoltaic effects when exposed to solar radiation to generate electricity. Photons become excited when exposed to solar radiation and then release electrons collected by wires to generate a direct current of electricity that most needs to be converted to alternating current using converters (Leggett, 2009). The produced energy can be stored using a storage device such as a battery or transmitted to load for utilisation. The device continues to generate energy as long as it is exposed to sunlight.

The photovoltaic effect was first discovered by a French scientist named Alexandre Edmund Becquerel in 1883. The scientist observed while experimenting with metal and electrodes. In the same year, the first photovoltaic cell from selenium wafers with about 1% efficiency was developed by an American inventor called Charles Fritts. In 1888, the first US patent for the photovoltaic cell was received by Edward Weston. In 1901, Nikola Tesla also received another US patent for the technology and a capable methodology utilising solar energy. In 1904, Albert Einstein published the theoretical basis of the photovoltaic effect, which earned him a Nobel Prize in 1923 (Akinyele et al., 2015). This critical development was advanced by a Polish scientist called Jan Czonchralski in the mid-twentieth century, who developed the silicon cell with 11% efficiency in 1958 at an unaffordable price (USD 1000/W) (Akinyele et al., 2015; Kalogirou, 2014). Baurzhan and Jenkins (2016) reported that the levelized cost of energy generated from the solar photovoltaic system was very high, but the cost has decreased and will continue to decline with the recent technological development. Also, Roche and Ifeanyi-Nwaoha (2017) stated that the solar photovoltaic system's cost drastically dropped by 60% between 2010 and 2015, and the cost of the technology is projected to drop further by 5.9% in 2015. The low cost of technology was due to the higher appetite for the solar photovoltaic electricity harvesting system and massive production (Sundaram et al., 2016). With this rapid declination of PV module prices, the technology has a promising future. However, despite this decline in photovoltaics' cost price, some developing countries like Nigeria are yet to utilise the technology's potential and continue to face energy starvation.

**Fig. 15:**
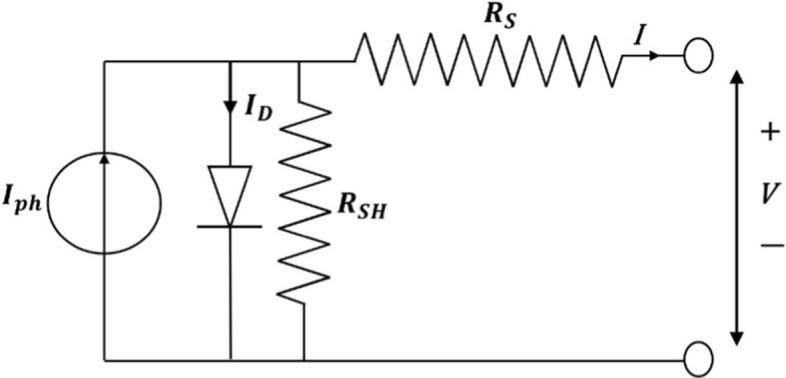
Circuit diagram of a typical solar PV cell (Kalogirou, 2014)

The photovoltaic effect occurs when an influx of solar energy hits a cell and gets absorbed by an atom's valence electron, creating pairs of electron holes. The level of electron energy increases according to the amount of photon absorbed. When the photon's energy is greater than the semiconductor bandgap, the electron with excess energy will escape to the conduction band, where it will have freedom of movement. Therefore, the absorption of photon releases an electron that can be collected by employing an electric field across the cell's back and front (Kalogirou, 2014). When conductors are attached to this electric field with positive and negative terminals, it forms an electrical circuit, and electrons are collected in form electrical current called photocurrent ($I_{{{\text{ph}}}} )$. In the absence of illumination, the cell remains inactive and does not produce current. However, if the terminals are connected to a significant external voltage source, the yield is referred to as dark or diode current ($R_{{\text{S}}} ).$ Figure 15 illustrates the circuit diagram of an individual cell showing series resistance inside the cell and shunt resistance ($R_{{{\text{SH}}}} )$ from the diode. Considering the aforementioned, the net current is given in Eq. (1) provided by Kalogirou (Kalogirou, 2014).

1 $$I = I_{ph} - I_{D} = I_{ph} - I_{O } \left. {\left\{ {\exp \left[ {\frac{{e(V + IR_{S} }}{{kT_{C} }}} \right]} \right. - 1} \right\} - \frac{{V + IR_{S} }}{{ R_{SH} }}$$

where $I_{O}$ is the dark saturation current that strongly depends on ambient temperature, *e* is the electronic charge given as $= 1.602 \times 10^{ - 19}$ J/V, *V* is the imposed voltage across the cell, *k* is the Boltzmann's gas constant given as $= 1.381 \times 10^{ - 23}$ J/K, and $T_{C}$ is the absolute cell temperature.

Even though exposure to temperatures and other environmental factors such as dust formation generally reduce photovoltaic technologies' performance with time, the device is highly reliable and can last for an extended period. Bugaje (1999) reported that a photovoltaic module has an average expected life span of 20–25 years. Conversely, a recent study by Sambo et al. (2014) shows that photovoltaic modules are expected to operate for a period of 30 to 35 years. Solar photovoltaic systems are categorised into first-generation, second-generation, and third-generation solar cells (Sundaram et al., 2016).

Silicon is a natural resource that is abundantly available across the globe. The first generation is based on silicon and accounts for more than 85% of the photovoltaics industry. This category of photovoltaic is further divided into three types, according to the design and quality of the silicon, which are: (i) crystalline silicon (c-Si), (ii) polycrystalline (pc-Si), and (iii) thin film (a-Si). These technologies have a high-efficiency rate, with the mc-Si having about 25% efficiency and pc-Si having about 21.5% efficiency (Akinyele et al., 2015; Sundaram et al., 2016). These technologies have been on the market for a long time, and their prices are rapidly declining.

The second generation is mainly based on thin film and was developed to optimise material consumption when developing a solar cell. These categories include Copper Indium Gallium Selenide (CIGS), Copper Indium Selenide (CIS), Cadmium Sulphide (CdS/CdTe), and Cadmium Telluride (CdTe) (Akinyele et al., 2015; Sundaram et al., 2016). These are thin layer semiconductors and consume less material when compared to crystalline photovoltaics. The conversion efficiency of these technologies is relatively lower than crystalline technologies. The lower efficiency conversion rate has limited the acceptance of the technology in the photovoltaic market.

Third-generation solar photovoltaic aims to reduce the cost of manufacturing by using more environmentally friendly materials. The technologies under this category are dye-sensitised PV, organic and polymer photovoltaics, nanomaterial-based PVs, and concentrated photovoltaics (Akinyele et al., 2015; Sundaram et al., 2016). Due to the mechanical flexibility and materials used, these technologies have lower efficiency than the second generation. These technologies are undergoing intensive research across the globe to improve their conversion efficiency.

**Fig. 16:**
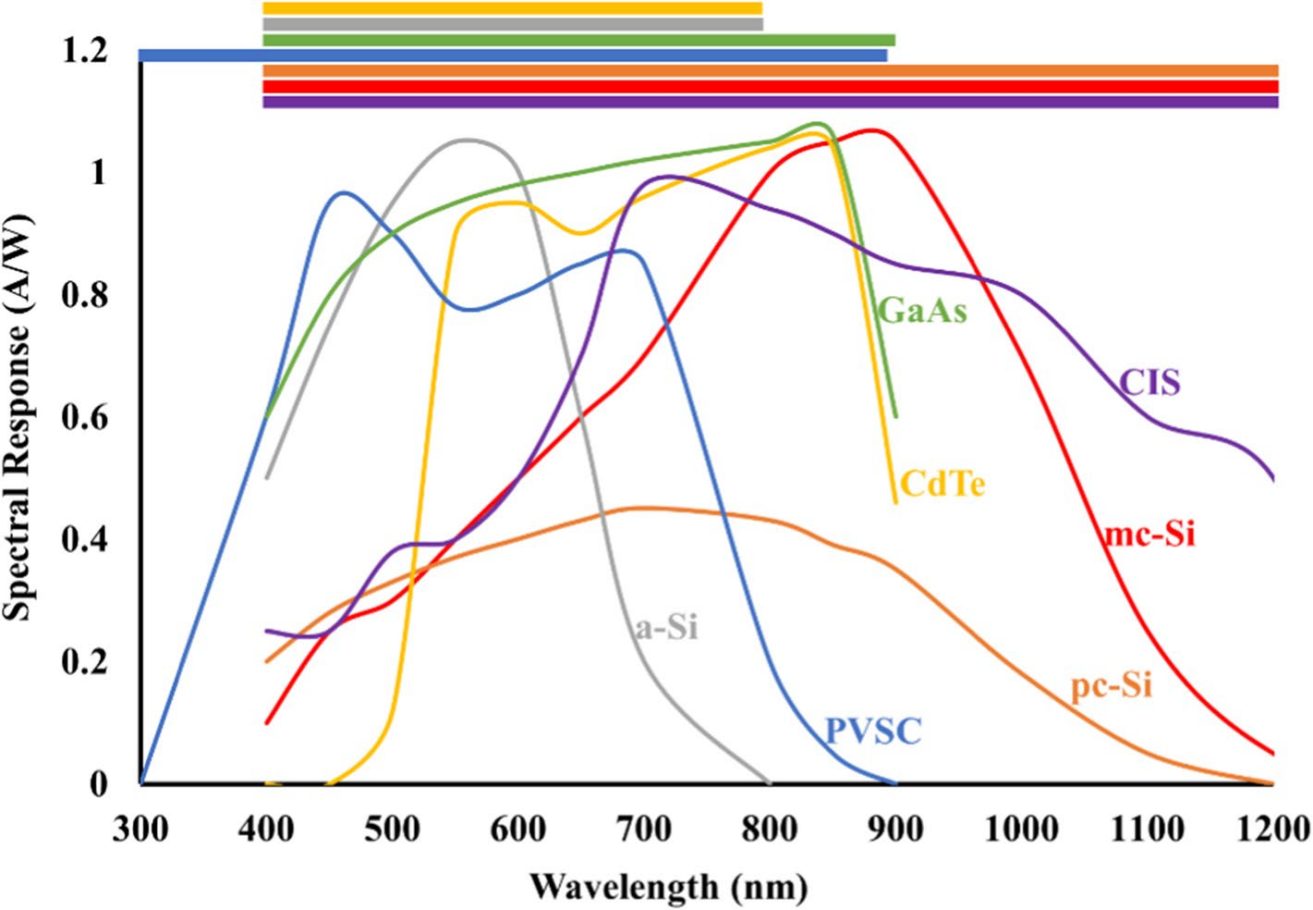
Variation of spectral response as a function of wavelength for various PV types

The categories of solar technologies mentioned above have been tested to generate electricity from free solar radiation, with no polluting (including air and noise pollution) effects. It can also provide electricity to remote areas which are not economically viable to extend the grid network. Each type of PV system mentioned above response to illumination in a wavelength range. Figure 16 illustrates the spectral response as a function wavelength.

With the abundance of solar energy potential in Nigeria, these technologies can be deployed to resolve the persisting energy crisis that has engulfed the nation for over two decades in rural electrification, residential and commercial buildings, water purification and pumping, street lighting, agricultural and industrial utilisation.

## Solar energy penetration in Nigeria

**Fig. 17:**
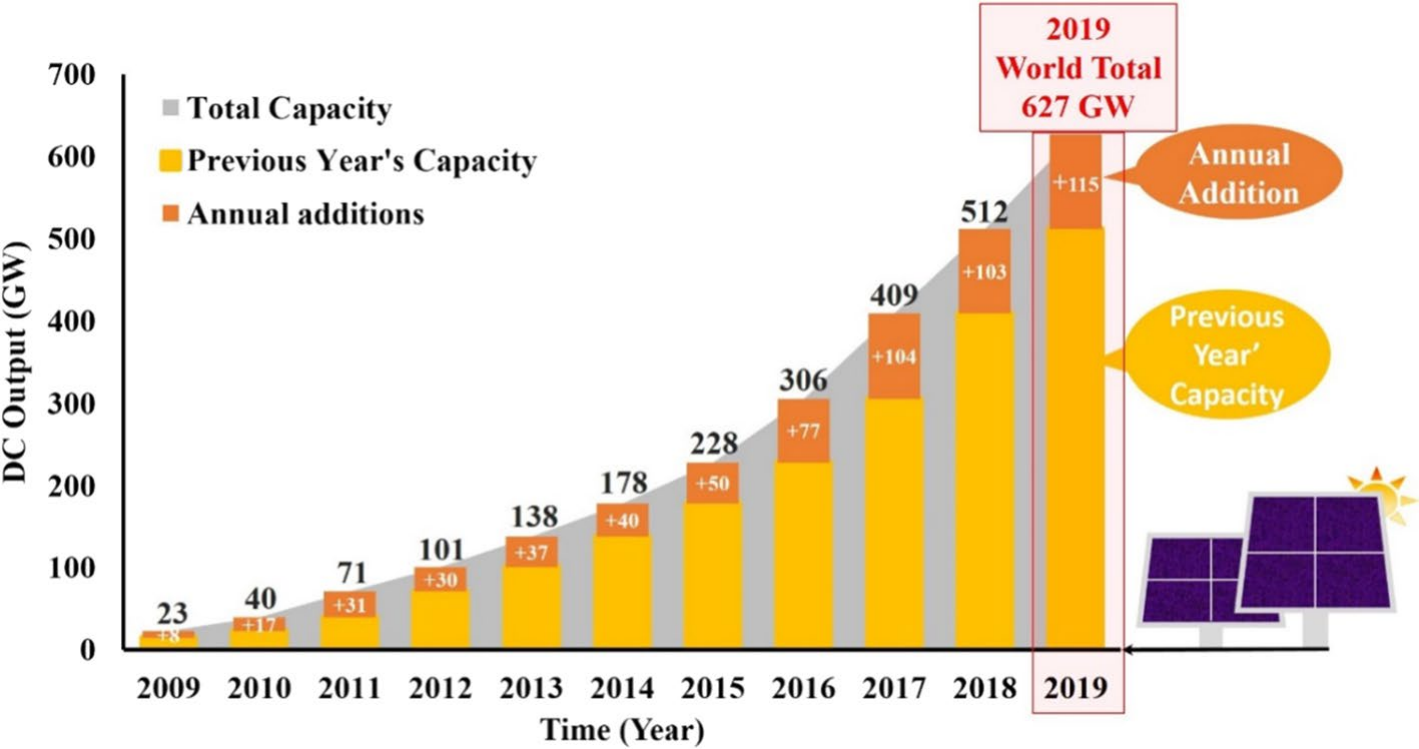
Solar energy installation ( adopted from REN21, 2,020)

The solar photovoltaic system is fast becoming the most reliable and promising clean energy technology globally. Ohunakin (2010) stated that solar energy systems have proven reliable for many decades in developed countries. According to REN21 (2020), in 2019, the global cumulative solar photovoltaic capacity was about 627 GW and net annual addition of about 115 GW of electricity in the same year. The annual increments for a decade from 2009 to 2019 are illustrated in Fig. 17. China alone accounts for 204.7 GW and has one of the largest solar PV plants (2.2 GWp with 202.8 MW/MWh storage) (IEA, 2021a, 2021b, 2021c).

Mohammed et al. (2017) and Ozoegwu et al. (2017) reported that most of Nigeria's available solar energy systems are limited to simple load electricity appliances in households and street lighting. In contrast, Sambo (2009) reported about 15 MW of isolated solar photovoltaic installation across the country. Also, many standalone installations of solar energy systems in the country are not adequately documented, resulting in the inaccurate data aggregation of solar PV penetration. Ozoegwu et al. (2017) stated that there are no accurate figures for the status of solar energy penetration in Nigeria, meaning there is no comprehensive statistical record such as a database for the deployment of solar energy technology in the country, which shows that the utilisation of solar energy is difficult to be ascertained highlighting that penetration might still be weak. Monyei et al. (2017) and Mohammed et al. (2017) concurred and supported this claim by stating that there is no exact and accurate data on the status of solar energy penetration in the country, but it has been established that so far, the solar energy has zero contribution to the national grid supply mix.

Nigeria's solar potential is very high but not adequately exploited. However, the Northern part of the country receives more solar radiation, and for that reason, it will be more suitable for solar electricity generation. If only 1% of the Northern Nigeria land area is made available for electricity generation from a solar energy system using 5% efficiency, about 333,480 MW of electricity may be generated at a 26% capacity factor (Sambo & Bala, 2012). As attractive as this may seem, the implementation concerns cannot be overlooked because it is practically impossible. Recently, in an attempt to integrate solar energy into the supply mix, the Federal Government approved a policy called NREEEP (National Renewable Energy and Energy Efficiency Policy), which intends to increase the percentage contribution of solar energy to the total energy supply mix to about 3% by 2020 and 6% by 2030 as the minimum contribution (NREEEP, 2015). Also, NASENI (National Agency for Science and Engineering Infrastructure) is a company that has been established to develop solar PVs in the country to promote and enhance the penetration of the technology. Aliyu et al. (2015) observe that solar energy penetration in the country is prolonged and further claim that technology's cost is 20 times higher than conventional fossil fuel. On the other hand, Mohammed et al. (2017) stated that solar energy technologies are now becoming relatively affordable as more households in the developed world are using the technology, and it is strategically vital for developing countries to adopt the technology as most of them are located in regions with high solar radiation.

Solar energy contribution to the nation's energy mix remains very negligible in the country when compared to its potential. This low penetration can be attributed to component failure, low awareness, technical problems, government policy, environmental factors, and vandalisation (Rui et al., 2018; Oyewumi and Oluiobi, 2015; Iledare, 2007). However, many local research centres are making giant strides in technology research and development to promote it to become a household commodity (Ilenikhena & Ezemonye, 2010). The solar energy system's application to supplement fossil fuel technologies or become part of the supply mix will ensure sustainable energy development, which has enormous advantages such as easy integration, zero noise and air pollution, replenishable resources, low maintenance cost, and a long operational life cycle (Luderer et al., 2019).

**Fig. 18:**
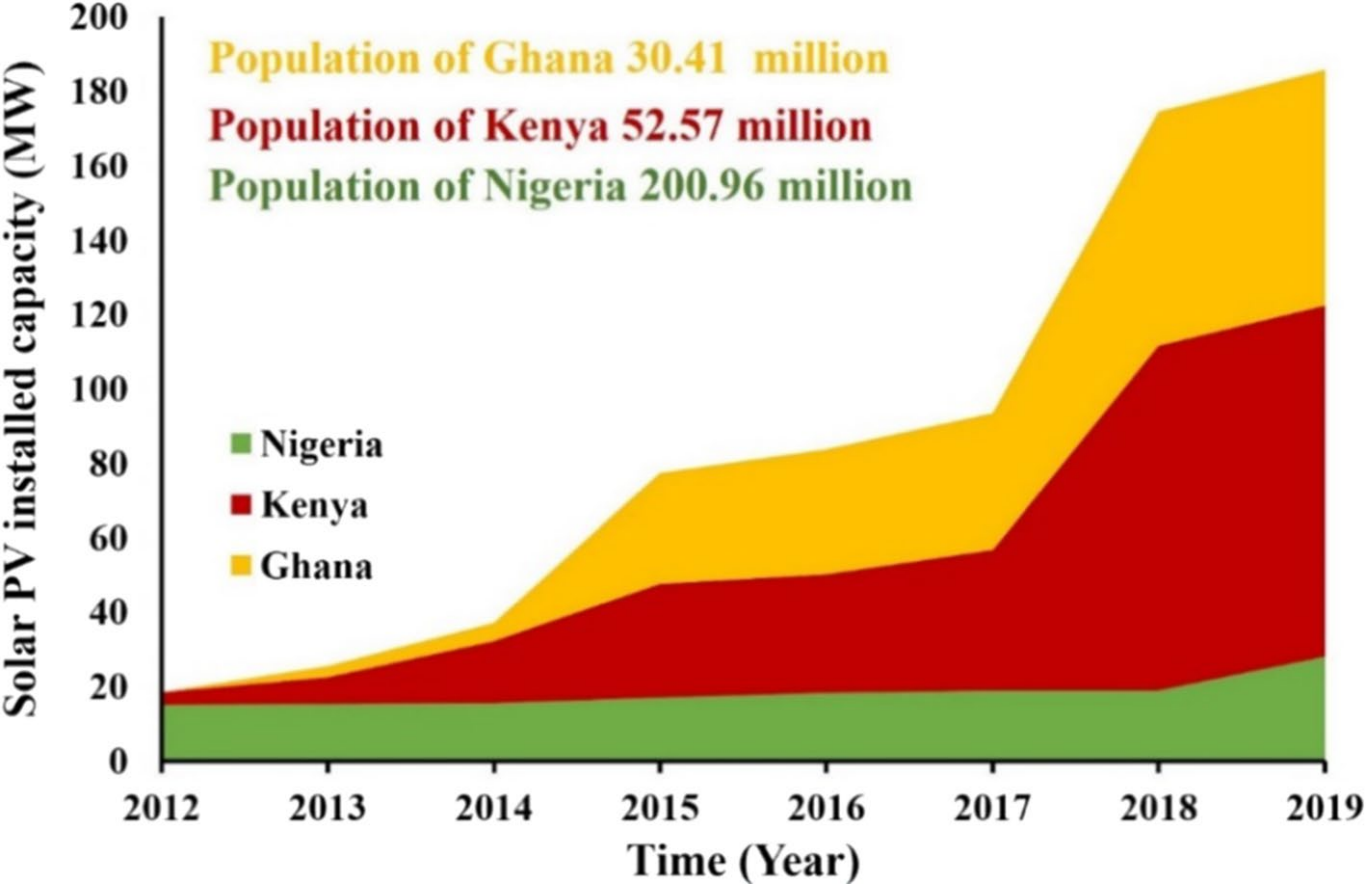
PV installed variation among three sub-Saharan countries (IRENA, 2020)

Ozoegwu et al. (2017) also stated that the integration of solar energy systems into Nigeria's national grid in the past is nil, currently is also nil, and future status may only be seen on memoranda of understanding. In contrast, some large solar farms were recently commissioned: 1 MW (Nigeria-Electricity-Hub, 2018), 7.1 MW in Bayero University Kano (Energy_Storage, 2019), and 1.2 MW in Usman dam (Off_Grid_Nigeria, 2017), Abuja. Also, several solar farms projects are under construction, such as 100 MW in Sokoto, 50 MW in Nasarawa State, 100 MW in Katsina State, 100 MW in Kaduna State, and 100 MW in Bauchi State (Evwind, 2017). A total of 27.96 MW PV installation has been reported towards the end of 2019 (IRENA, 2018). However, the amount is meagre than other developing countries in the region, illustrated in Fig. 18. The country with less energy demand a smaller population than Nigeria has high PV penetration rates even though Nigeria started using technology.

However, there are a large number of ongoing on-grid solar energy projects in the country, such as 1 MW solar project, which the French oil company launched in Northern Nigeria (Aliyu et al., 2017); 58 projects embarked by the Energy Commission of Nigeria (E.C.N.), 3 MW of utility-scale solar photovoltaic (PV) projects by Delta State Government; Zamfara State solar electrification, 600 projects by Rural Electrification Agency; Niger State 300 MW solar power plant and the Anjeed Kafanchan 15 MW among others. The Nigerian government signed an M.O.U. (memorandum of understanding) with a US-based company to provide 1200 MW electricity from solar PV projects within two years at the cost of over USD 2 billion (Mas’ud et al., 2015). Recently, Nigeria has launched a USD 75 m project to use solar PV in generating electricity for its citizens (Power_Technology, 2020).

The significance of solar energy development and integration into the supply mix is clear and well recognised. Aliyu et al. (2015) hold the view that solar energy technology has an enormous advantage to resolve the energy poverty of small settlements in remote areas that are not connected to the national utility grid and provide other necessities such as rural clinic electrification, water supply, traffic lighting, and school lighting. Ohunakin et al. (2014) also point out why solar energy should be integrated into the country's supply mix. These reasons are: solar energy can reduce carbon footprint, provide energy security, improve supply, improve rural electrification, and create job opportunities. Nigeria must bridge the gap between demand and supply by using renewable energy technology such as solar to overcome its present electricity crisis and pursue economic prosperity. However, security issues, infrastructural deficit, corruption, vandalism, local content, underfunding, institutional, and human capital development are some challenges that the technology’s penetration is encountering (Rui et al., 2018; Gian, 2017; Oyewunmi and Olujobi, 2015; Iledare, 2007).

### Challenges facing solar technology penetration in Nigeria

This paper also highlights some challenges confronting solar energy penetration in the country, and without good penetration, sustainable development would be compromised. Ohunakin et al. (2014) highlighted the drivers and barriers of solar energy penetration in the country, such as lack of public awareness, government policy, theft, and solar technology vandalism. Akinboro et al. (2012) also reviewed standalone PV installation for Nigeria's residence and industries. Researchers highlighted the problems and recommended a possible solution. They highlighted the issues confronting solar installation in Nigeria and recommended using solar PV in a hybrid to avoid blackouts. Similarly, in a research paper, Sambo and Bala (2012) discussed solar energy penetration in Nigeria and highlighted solar energy penetration status and challenges. Based on the papers mentioned above, a summary of the challenges confronting the penetration of solar technology is categorised into three groups as follows:

### Technical

#### Present level of research and development

There is no in-depth research on the challenges confronting solar energy penetration in the country, and most local research centres are not focusing their efforts on that. There is no state-of-the-art research on technological development related to solar energy in the country, causing slow penetration of the technology (small scale or large scale) due to funding constraints; thus, the technology is yet to become widely available. The country has inadequate human capacity building and related training in solar energy development, installation, and maintenance (Mohammed et al., 2017).

#### Technology and equipment fabrication

Considering solar irradiance potential in Nigeria, the country has a low level of fabrication technology rating. Several companies such as Lumos, Auxano Solar, Solmont Technologies, and Sunhive strive to establish solar photovoltaic assembling lines in the country. As earlier established, NASENI is the only indigenous establishment in the country recorded to be manufacturing solar photovoltaic (NASENI, 2011); otherwise, most solar photovoltaic systems identified in the country were imported, and most of them are found to be substandard products (Ohunakin et al., 2014).

#### Component failure

Several solar energy technologies in the country tend to drop in performance or become inefficient shortly after installation (Ikem et al., 2016). This component failure is mainly caused due to use of substandard technology and/or environmental and climate effects such as dust formation on the photovoltaic panel (Oji et al., 2012). It makes the users turn away from using the devices and gives them a negative image.

#### Technical problem

Fundamental design problems such as suitable geographical site identification, load profile determination, intermittency of resources, and the inclination angle are ignored by inexperienced personnel engaged in a solar installation in the country (Sambo et al., 2014). The country has few professionals capable of designing and installing the appropriate setup for residential, commercial, and industrial buildings but are primarily engaged with other solar energy-related activities, allowing inexperienced personnel to handle the installations. There is the insufficient technical knowledge of developing energy management systems to integrate solar energy technology with the national grid.

Transmission Company of Nigeria (TCN) is wholly owned by the government and is affected by inconsistent and inadequate funding. As earlier stated, outdated facilities in its network characterise TCN incapable of accommodating the energy generated; additional solar farms might face the same challenges. The transmission company cannot meet its expansion plan as it is engulfed with debt and cannot generate sufficient funds to tailor it to purchase new equipment (Ibukun & Adebayo, 2021).

#### Poor maintenance

Nigerians have a very poor maintenance culture. Many solar photovoltaic projects in the country fail because of low maintenance (Sambo et al., 2014). The solar photovoltaic installations around the country are not kept clean, and with dust particles, birds' droppings, and other materials on top of the panel, the system's performance tends to decrease. Chanchangi et al. (2020) reported how dust activities in the region significantly affect the PV system performance and hinder its penetration and utilisation. The dust has been reported in other regions to significantly affect the performance of PV technology (Chanchangi et al., 2020; Gupta et al., 2019).

### Economical

#### Affordability

As earlier stated, most Nigerians live below the poverty line, and most of them reside in rural areas where electricity access is low; as such, most of them cannot afford to acquire a complete solar energy setup because of the price. The price of solar energy technology and its installation cost is still considered expensive in this part of the world, especially among the rural population (Giwa et al., 2017; Ikem et al., 2016; Mohammed et al., 2017; Oji et al., 2012).

#### Cost of generation

The initial cost of installing solar photovoltaic technology is very high compared with other energy harvesting systems such as diesel generators in the short run. However, the technology is far cheaper in the long term. The affordability issue by low-income earners will appear, as most people living without access to the national grid cannot afford the technology (Ikem et al., 2016).

#### Debt

The energy sector faces increasing illiquidity, and massive losses as generated electricity cannot be quickly sold since most of the population bypasses electricity from the grid to avoid payment. This scared up potential investors as it is difficult to profit from the investment (Babatope & Solomon, 2020).

Energy companies are in serious debt as the sector has been impacted by liquidity and solvency, restricting them from meeting up their targets. However, the Central Bank of Nigeria (CBN) is working to restructure the debt policy on energy sectors, thereby preventing the companies from filing bankruptcy (Babatope & Solomon, 2020).

Lack of payment discipline by Nigerians is a factor that affects the existing supply system in the country as consumers are refusing to pay their bills. It was reported in 2020 that Nigerians owe N273.42bn electricity debt (Nwachukwu, 2021). Internationally, indebtedness is increasing by the day as customers such as Mali, Niger, and Togo owe massive amounts, with the total owed by the three countries to be around USD 69 m (Jeremiah, 2020)**.**

### Policy

#### Government policy

There have been policy implementation U-turns over the years by the Nigerian government regarding integrating solar energy in the national supply mix due to government ignorance. However, the NREEEP policy that aims to contribute about 3% in 2020 to about 6% in 2030 of solar energy to the national supply mix is available (Giwa et al., 2017), but has not been fully implemented; as the government has not yet fully implemented some of the sub-policies such as a subsidy programme, incentives and any other palliative mechanism to encourage and attract potential investors to the sector (Giwa et al., 2017; Ikem et al., 2016; Mohammed et al., 2017). There is a lack of importation taxation waivers in the power sector. According to Ohunakin et al. (2014), import duty for solar technology is about 21%, which is side compared to petroleum oils and oils obtained from bituminous minerals, which is crude of 5%. Policy instability has the potential investors to doubt the commitment credibility of the government, which position the private sector investment to be at a high-risk level in the country (Roy et al., 2020).

#### Lack of awareness

The inadequate dissemination of information related to the enormous solar energy potential in the country and lack of awareness related to immense solar PV benefits leads to scepticism in the majority about the deployment of solar energy technology for energy generation (Giwa et al., 2017; Mohammed et al., 2017; Ohunakin et al., 2014; Oji et al., 2012). There is also a lack of awareness regarding climate change and the consequences of GHG emissions from fossil fuel utilisation in electricity generation.

#### Theft and vandalism

Most especially standalone systems, solar energy technologies are prone to theft and vandalism (Mohammed et al., 2017). The area with high energy demand in the country is more prone to theft and vandalism of solar energy technologies due to insecurity and insurgency (Ohunakin et al., 2014).

Vandals remove or cut some transmission cables or infrastructures in protest against government policy or for stealing purposes. This poses a significant concern for the transmission of generated electricity from solar energy infrastructures. The activities of vandals significantly impact the transmission infrastructure and have persisted over two decades, leading to the continuous breakdown that negatively impacted the living standard of Nigerians (Ibukun & Adebayo, 2021).

#### Security instability

Security is paramount for the penetration of renewable energy technology. Security instability has been a significant concern in Nigeria, with the activities of terrorists, kidnappers, insurgents, militants, and bandits across the country (BBC, 2021); it is posing a threat to enabling investment environment even though there is a substantial potential market due to population and demand gap. The insecurity directly affects all aspects that could enhance the penetration of renewable energy technology in the supply mix. Investors would require a secured region to commit; similarly, installed infrastructure must be secured over a long period for its return investment. Local research and development could only be achieved in a stable and secured environment.

Ohunakin et al. (2014) reported that kidnapping and killings across the country, particularly in Northern Nigeria, have impeded the construction of some power plants and other infrastructure that could promote the country's penetration of renewable energy technology. This is assumed to stymie potential threats to the future development of the solar energy infrastructure, especially at a large scale. Miftahu and Oruonye (2020) reported that the country is overwhelmed by security challenges from terrorism, insurgency, conflicts, community-based militias, and bandits, making it a high-risk and vulnerable region that affects all kinds of investment.

## Discussion and policy recommendation

The rapid growth of Nigeria's population has created a wide gap between the demand and supply of electricity, and the overdependence on fossil fuel has severe adverse socio-economic, environmental, and health effects. The country suffers from an unstable, unreliable, and inadequate electricity supply. Energy poverty is very high, yet the enormous abundant renewable energy in the country is still untapped. The economic and environmental benefits of the PV technology have made the system the most sustainable clean energy solution to enable developing countries to achieve the UN’s SDG-7 target. This has led countries such as Nigeria to invest in solar energy to increase the penetration of renewable energy in their supply mix and reduce global warming. However, as mentioned in this report, the technology faces several challenges, one of which appears to be a technical problem.

Reviewing the related literature emphasises that PV systems are developing at an incredible increasing rate. The reason is the favourable solar conditions and new supporting legislation. However, dust activities act as substantial barriers to PV development. A thorough literature review was also provided to understand these activities, origins, and frequencies.

The main aim of this study was to review the Nigerian scientific research concerning energy deficiency and the potential of renewable energy in the country, focusing on solar energy technologies and their development, penetration, and the problems hindering penetration. In conclusion, several challenges for further research were identified, and the recommendations in these regards can be summarised as follows.

Nigeria has abundant renewable energy resources readily available for tapping and integrating to supply mix with a more promising future in solar PV. Therefore, the government should speedily prioritise solar PV development across the country.

Research and development need additional funding from both the government and private sectors to examine various solar PV technology performances across the country and provide benchmarks for future innovations that would suit the regional conditions.

Regulators such as standard organisations should ensure that substandard renewable energy products are prevented from penetrating the local market. All renewable energy products should be subjected to rigorous tests for certification before being allowed into the local market.

More technical personnel across the country should be trained on the standard solar photovoltaic installation and maintenance procedures. Vocational training could be provided to qualified and desired candidates to reduce wrong PV installation practices.

An overhaul of the transmission company should be conducted, starting with an audit to forecast the peak demand and possible peak generation. New infrastructures should be installed to accommodate maximum potential generation from various renewable sources, especially solar PV.

PV installation maintenance required background research across the country to investigate the impact of various parameters such as soiling (dust accumulation), temperature, shading, and accidental damages. Moreover, their influence concerning the technology's performance, since most of the parameters are site-specific. This would ease determining a cost-effective approach and the required frequency for optimum PV yield performance. Practical experiment concerning the impact of the parameters mentioned above is relatively understudied; it opens up a wider gap in the research and development (R&D) for the solar PV community.

Energy losses from PV due to dust are an issue that cannot be ignored and can be an obstacle to achieving renewable energy targets in Nigeria. In this context, this recommended further investigation concerning an approach that could sustain and maintain a certain level of performance over the device's life cycle.

Since the price of solar photovoltaic is dropping, other related components should also be subsidised to assist low-income earners in acquiring the technology. Another alternative is that a tax incentive policy should be implemented to reduce the cost prices of the technology. The institution should be encouraged to offer interest-free loans for solar energy deployment. The government should also offer incentives such as subsidies, taxation waivers, and investment loans in the sector. These financial supports will reduce the initial high cost of solar technologies in the country.

Policy U-turn should be avoided, and development continuity should be embraced to complete energy projects fully.

The Nigerian government must pursue the documented Nigerian Renewal Energy and Energy Efficiency Policy (NREEEP) for incorporating renewable energy in the supply mix, diversifying and increasing the supply. The government should also try to implement the policy and its recommendations since the 3% target of solar energy inclusion in the supply mix set for 2020 has not been met, and 2030 is fast approaching.

More awareness campaigns should be developed at all levels through seminars, workshops, promotional exercises, and media adverts to highlight and publicise the benefits and opportunities associated with solar technology to encourage people to adopt the technology. Both private and government sectors should conduct an awareness campaign.

Appropriate measures such as anti-theft screws, cementing photovoltaic base, and inscription should be employed to prevent theft and vandalisation of solar energy technologies. Continuous surveillance improvement by law enforcement agencies will also assist in preventing both theft and vandalisation.

Finally, solar energy remains the most viable option to solve the persisting energy crisis in Nigeria as it can supply electricity from off-grid and remote areas. It can reduce both the pressure mounted on fossil fuel and has a limited negative impact on our ecosystem and its inhabitants and, as such, should be urgently exploited. This research study is limited to secondary information as no primary data were collected; however, it is highly recommended to investigate the country's actual penetration level of solar energy technology. This might require a considerable resource but would be very useful for planning and other data application. Reviewing the Nigerian energy sector and focusing on solar PV potential revealed several challenges, as presented in the previous section. This study recommended a thorough investigation of the challenges, particularly the technical problem and PV maintenance.

## Conclusion

The rapid growth of Nigeria's population has created a wide gap between the demand and supply of electricity, and the overdependence on fossil fuel has severe adverse socio-economic, environmental, and health effects. This study highlighted that the country suffers from an unstable, unreliable, and inadequate electricity supply. Energy poverty is very high, yet the enormous abundant renewable energy in the country is still untapped. The economic and environmental benefits of the PV technology have made the system the most sustainable clean energy solution to enable developing countries to achieve SDG-7. This has led them to invest in solar energy to increase the penetration of renewable energy in their supply mix and reduce global warming.

This study reviewed Nigeria's energy crisis and transition to clean energy, focusing on solar energy potential and penetration, opportunities, and challenges. The review findings highlighted several challenges that were found to be hindering the penetration of the technology in the region. Policy recommendations were provided, and research gaps were presented for further studies to ensure an increase in penetration and sustenance of the performance of the technology over its lifetime and enhance acceptance. One of the main areas of study identified was the impact of dust accumulation on PV performance since it adversely affects it. This area has been underestimated and understudied in the regions. It is recommended that thorough investigations are conducted on the impact of dust accumulation on PV performance to improve solar PV technology penetration and reduce GHG emissions in Nigeria. A significant number of studies were thoroughly reviewed, and this report can serve as a guide for renewable energy researchers, the general public, potential inventors, the government, and the science community. Nigeria needs sustainable green energy to develop and sustain its economy.

## Data Availability

The datasets used and/or analysed during the current study are available from the corresponding author on reasonable request.
